# Identification and validation of crucial lnc-TRIM28-14 and hub genes promoting gastric cancer peritoneal metastasis

**DOI:** 10.1186/s12885-023-10544-8

**Published:** 2023-01-23

**Authors:** Chao Dong, Fujuan Luan, Wenyan Tian, Kaipeng Duan, Tao Chen, Jiayu Ren, Weikang Li, Dongbao Li, Qiaoming Zhi, Jin Zhou

**Affiliations:** 1 Department of General Surgery, The First Affiliated Hospital of Soochow University, Suzhou, 215006 China; 2 Department of Gastroenterology, The First Affiliated Hospital of Soochow University, Suzhou, 215006 China

**Keywords:** RNA-seq, WGCNA, lncRNA, Gastric cancer, Peritoneal metastasis

## Abstract

**Background:**

Gastric cancer peritoneal metastasis (GCPM) is an important cause of cancer-related deaths worldwide. Long non-coding RNAs (lncRNAs) play a key role in the regulation of GCPM, but the underlying mechanisms have not been elucidated.

**Methods:**

High-throughput RNA sequencing (RNA-seq) was performed on four groups of clinical specimens (non-metastatic gastric cancer primary tumor, adjacent normal gastric mucosal tissue, gastric cancer primary tumor with peritoneal metastasis and adjacent normal gastric mucosal tissue). After sequencing, many lncRNAs and mRNAs were screened for further Weighted Gene Co-expression Network Analysis (WGCNA). GCPM-related hub lncRNAs and genes were identified by cytoHubba and validated by Quantitative real-time PCR (qRT-PCR), Receiver operating characteristic curve (ROC) analysis and Kaplan-Meier survival analysis. GO, KEGG and GSEA showed GCPM-related pathways. Correlation analysis revealed the potential relationship between hub lncRNAs and genes.

**Results:**

By analyzing lncRNA expression data by WGCNA, we found that blue module was highly correlated with GCPM (r = 0.44, *p* = 0.04) and six lncRNAs involved in this module (DNM3OS, lnc-MFAP2-53, lnc-PPIAL4C-4, lnc-RFNG-1, lnc-TRIM28-14 and lnc-YARS2-4) were identified. We then performed qRT-PCR validation of gastric cancer specimens and found that the expression of lnc-RFNG-1 and lnc-TRIM28-14 was significantly increased in gastric cancer tissues with peritoneal metastasis. Kaplan-Meier survival analysis showed shorter overall survival time (OS) for gastric cancer patients with high expression of lnc-TRIM28-14. Receiver operating characteristic curve (ROC) analysis showed that lnc-TRIM28-14 could improve the sensitivity and specificity of GCPM diagnosis. In addition, we identified three key mRNAs (CD93, COL3A1 and COL4A1) associated with gastric cancer peritoneal metastasis through WGCNA analysis and clinical specimen validation. Moreover, there was a positive correlation between lnc-TRIM28-14 and the expression of CD93 and COL4A1 in gastric cancer peritoneal metastasis, suggesting a regulatory relationship between them. Subsequent GO, KEGG and GSEA analysis suggested that ECM-receptor interaction and focal adhesion were the hub pathways of GCPM.

**Conclusion:**

In summary, lnc-RFNG-1, lnc-TRIM28-14, CD93, COL3A1 and COL4A1 could be novel tumor biomarkers and potential therapeutic targets for GCPM.

**Supplementary Information:**

The online version contains supplementary material available at 10.1186/s12885-023-10544-8.

## Background

Gastric cancer (GC) is a malignant disease posing a serious threat to human health. Over one million new gastric cancer cases were reported in 2020, resulting in an estimated 769,000 deaths worldwide [1]. Currently, it has the fifth highest incidence and accounts for the fourth highest mortality among all cancers globally. Recurrence of peritoneal metastasis is one of the leading causes of death in patients with advanced gastric cancer. Peritoneal metastasis refers to a form of cancer metastasis caused by the direct growth of cancer cells in the primary tumor of gastric cancer through blood, lymph or peritoneum [2]. Nearly 20% of gastric cancer patients were diagnosed with peritoneal metastasis before or during surgery, and more than 50% of patients with T3 and T4 stages developed peritoneal metastasis after radical resection. The higher the degree of peritoneal metastasis, the shorter the survival time [3]. Consequently, searching for novel treatments for gastric cancer peritoneal metastasis is of utmost importance.

Long non-coding RNAs (lncRNAs) are a class of RNAs over 200 nucleotides in length and are generally considered to have no coding capacity [4, 5]. In recent years, lncRNAs have been found to play multiple roles in the initiation, development and metastasis of cancers [6]. Specifically, Lnc030 has been reported to be involved in maintaining cancer cell stemness and promoting cancer initiation and progression in breast cancer [7]. LncRNA H19 has also been found to play a crucial role in bone metastasis of hepatocellular carcinoma [8]. In gastric cancer, lncRNA GCMA was found to promote growth, invasion, and metastasis through a competing-endogenous-RNAs pattern [9].

Weighted Gene Co-expression Network Analysis (WGCNA) is a more systematic and comprehensive R package for high-dimensional data processing than conventional differentially-expressed-gene analysis. Using WGCNA, it is possible to identify and screen key biomarkers and co-expressed gene modules [10]. Zhang et al. identified methionine sulfoxide reductase B3 (MSRB3) as a key gene up-regulated in peritoneal metastasis of gastric cancer using WGCNA [11]. Zhang et al. identified estrogen receptor 1 (ESR1), histone deacetylase 1 (HDAC1) and clathrin heavy chain (CLTC) associated with the occurrence of stomach adenocarcinoma using integrated bioinformatics analysis [12]. However, to our knowledge, there is no report identifying key lncRNAs for gastric cancer peritoneal metastasis using WGCNA.

In this study, we performed whole-transcriptome sequencing on tissue samples of gastric cancer peritoneal metastasis, and identified GCPM-related lncRNAs (lnc-RFNG-1 and lnc-TRIM28-14) and mRNAs (CD93, COL3A1, and COL4A1). Intriguingly, lnc-TRIM28-14 was positively correlated with the expression levels of CD93 and COL4A1 in gastric cancer peritoneal metastasis, suggesting a regulatory relationship between them. Consequently, we hypothesized that lnc-TRIM28-14, CD93 and COL4A1 could regulate GCPM and could be novel tumor markers and potential therapeutic targets for GCPM.

## Materials and methods

### Tissue specimens

**Fig. 1:**
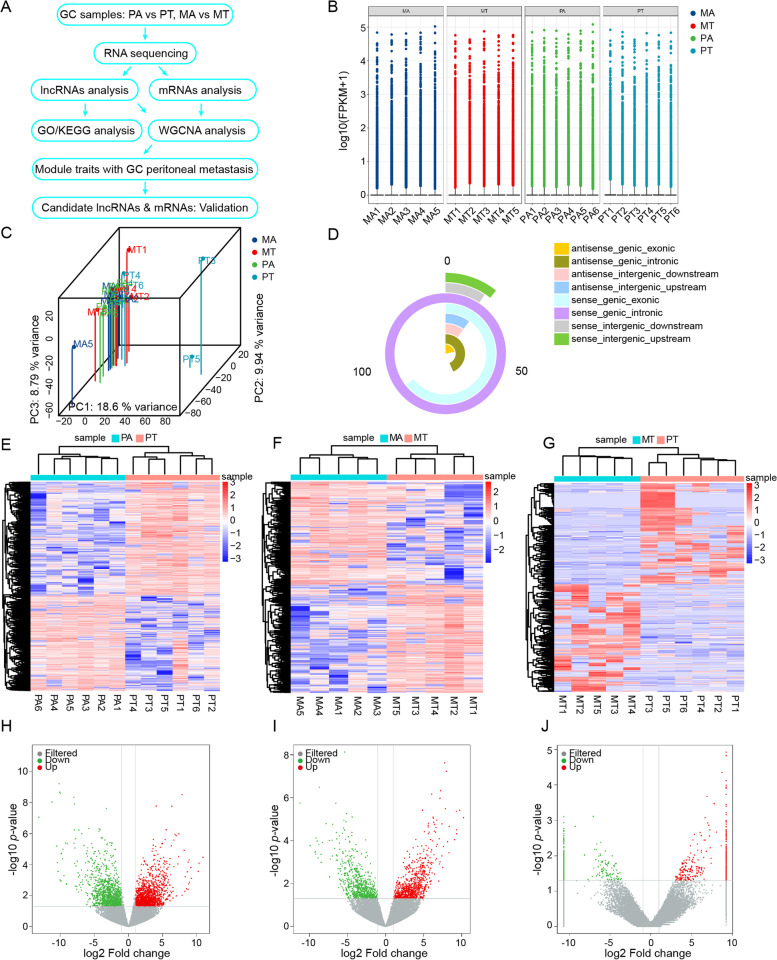
Expression profile of lncRNAs in gastric cancer tissues and adjacent tissues. **A** RNA sequencing and analysis flowchart. **B** Boxplot of FPKM values of lncRNAs in each sample. **C** Principal component analysis based on the expression of lncRNAs. **D** Novel lncRNAs classification. **E**–**G** Heatmap showing lncRNAs expression in PTvsPA **E**, MTvsMA **F**, MTvsPT **G**. **H**-**J** Volcano plot showing the lncRNAs expression pattern in PTvsPA **H**, MTvsMA **I**, MTvsPT **J**

The study design process is shown in Fig. 1A. Primary GC tissues and the corresponding non-cancerous adjacent tissues were collected from 90 patients who underwent gastric resection for GC without neoadjuvant therapy at the First Affiliated Hospital of Soochow University from 2010 and 2020. Among them, four groups of clinical specimens (non-metastatic gastric cancer primary tumor (PT) and adjacent normal gastric mucosal tissue (PA), gastric cancer primary tumor with peritoneal metastasis (MT) and adjacent normal gastric mucosal tissue (MA)) were used for whole-transcriptome sequencing (OE Biotech, Shanghai, China). The remaining samples were stored at -80 °C for qRT-PCR.

All patients provided written informed consent. This study was performed in accordance with the principles of the Declaration of Helsinki. The Ethics Committee of the First Affiliated Hospital of Soochow University approved this study (approval number: 2020381).

### High-throughput RNA sequencing

Total RNA was extracted using the mirVana miRNA Isolation Kit (Ambion, Austin, TX, USA) following the manufacturer’s protocol. RNA integrity was evaluated using Agilent 2100 Bioanalyzer (Agilent Technologies, Santa Clara, CA, USA). Samples with RNA Integrity Number ≥ 7 were selected for subsequent analysis. Sequencing was done by OE Biotech (Shanghai, China) and the normalized expression matrix is in the attached table (Table S1).

### LncRNA prediction, differential screening analysis and functional Analysis

The results of alignment with the reference genome were stored in a bam file. Using Stringtie software to assemble reads, the new transcript was spliced. Then, candidate lncRNA transcripts were selected by comparing gene annotation information of the reference sequence produced using Cuffcompare software. Finally, transcripts with coding potential were screened out using CPC, CNCI, Pfam and PLEK to obtain predicted lncRNA sequences. Sequencing reads from each sample were aligned with known lncRNA sequences and lncRNA prediction sequences using bowtie2.The eXpress tool was used for quantitative gene analysis to obtain FPKM value and counts value (the number of reads for each gene in each sample).

The estimateSizeFactors function of the package DESeq (2012) in R was used to normalize the counts, while the nbinomTest function was used to calculate *p*-value and flodchange values for comparison of differences. Transcripts with *p*-values ≤ 0.05 and flodchange ≥ 2 were selected, and lncRNA GO and KEGG enrichment were analyzed using Hypergeometric Distribution Test [13].

### Construction of WGCNA

A co-expression network was constructed with lncRNAs or genes selected using the WGCNA algorithm package in R project (version 4.0.3, https://www.r-project.org/). Average connectivity and scale independence analysis of modules with different power values were performed using gradient test (power value ranging from 1 to 20). Appropriate power value was determined when the scale independence value was equal to 0.8. WGCNA algorithm was then used to construct co-expression modules and extract gene information in each module. For module detection, hierarchical clustering was used to produce a hierarchical clustering tree (dendrogram) of genes with the function hclust based on dissTOM. The Dynamic Tree Cut method was used for branch cutting to generate modules and a relatively large minimum module size of minClusterSize = 30. Modules with similar expression profiles were merged when the correlation of module epigengenes was greater than 0.70 [11].

### Quantitative real-time PCR (qRT-PCR)

Total RNA was isolated from GC tissues or cultured cells using a Trizol (Invitrogen, USA) standard protocol. The integrity, quantity, and purity of RNA were determined using NanoDrop 2000c Spectrophotometer (Thermo Scientific, Wilmington, USA). Briefly, 1 µg of total RNA was reverse transcribed using All-In-One 5 × RT MasterMix (ABM, Canada). Real-time quantitative PCR reactions were then performed on an ABI ViiA7 Sequence Detection System (Life Technologies, USA) using SYBR Green Master Mix (Novoprotein, Shanghai, China). Relative gene expression levels were analyzed using 2^−ΔΔCt^, where Ct was the cycle threshold number normalized to GAPDH [14]. The primers used are shown in Table S2. PCR products were separated by 2% agarose gel electrophoresis under 100 V for 1 h.

### Functional enrichment analysis

Gene Ontology (GO) and Kyoto Encyclopedia of Genes and Genomes (KEGG) pathway analysis were performed based on standard settings in the DAVID database (https://david.ncifcrf.gov). GO terms and KEGG pathways were ranked by *p*-value (or -log10 (*p*-value)) with *p* < 0.05 as the cutoff criteria. Gene set enrichment analysis (GSEA) was performed with GSEA software (version 4.0.3, http://www.broadinstitute.org/gsea).

### ROC curve and Survival analysis

The area under ROC curve (AUC) was calculated to quantify ROC. Sensitivity and specificity were calculated for optimum cutoff values. Survival curves were calculated using the Kaplan-Meier (KM) method and compared with the log-rank test. Univariate and multivariate Cox regression analyses were used to analyze prognostic value of clinicopathological features.

### Statistical analysis

SPSS 26.0 software (IBM Corp.) and GraphPad Prism 9 were used for all statistical analyses. Continuous data were presented as means ± standard error of the mean and differences between the experimental groups were analyzed using Student’s t-test. Correlation analysis was carried out using Pearson or Spearman test. The relationship between expression of hub biomarkers and other clinicopathological features was evaluated using suitable Chi-square tests. All statistical tests in the present study with *p* < 0.05 were considered statistically significant.

## Results

### Identification of lncRNAs associated with gastric cancer peritoneal metastasis

To identify lncRNAs associated with GCPM, we first performed RNA-seq using four sets of clinical specimens. The expression levels of lncRNAs were normalized using FPKM. Boxplot results showed no difference in expression levels of lncRNAs in each group (Fig. 1B). The Principal Component Analysis results showed that the sample dispersion in each group was small (Fig. 1C). In total, 425 new lncRNAs were predicted, with a total length of 1,019,124 bp and an average length of 2397 bp. The main type of lncRNA was sense_genic_intronic (Fig. 1D).

Further by screening differentially expressed lncRNAs (DElncRNAs) with |log_2_FC|> 1 and *p* < 0.05, the heat map and volcano plot revealed 2718, 1487 and 719 up-regulated or down-regulated lncRNAs in the PT vs. PA group (Fig. 1E, H), MT vs. MA group (Fig. 1F, I) and MT vs. PT group (Fig. 1G, J), respectively. These results confirm the presence of several abnormally expressed lncRNAs in the process of gastric cancer peritoneal metastasis, suggesting that they play an important role in regulation of GCPM.

### Ectopic lncRNAs associated with GCPM are involved in important biological processes

To evaluate the functions of DElncRNAs, GO and KEGG pathway analyses were performed. In PT vs. PA group, 3'-UTR-mediated mRNA stabilization, melanosome and translation activator activity were the most significantly enriched GO terms in biological process (BP), cellular component (CC) and molecular function (MF), respectively (Fig. S1A). Maturity onset diabetes of the young was the most significantly enriched KEGG pathway (Fig. S1B).

In MT vs. MA group, regulation of T cell anergy, apical tubulobulbar complex and 17-alpha,20-alpha-dihydroxypregn-4-en-3-one dehydrogenase activity were the most significantly enriched GO terms in BP, CC and MF, respectively (Fig. S1C). Mucin type O-glycan biosynthesis was the most significantly enriched KEGG pathway (Fig. S1D).

In MT vs. PT group, positive regulation of cell adhesion molecule production, proteasome accessory complex and signaling pattern recognition receptor activity were the most enriched GO terms in BP, CC and MF, respectively (Fig. S1E). Ether lipid metabolism pathway was the most significantly enriched KEGG pathway (Fig. S1F).

### lncRNA co-expression network construction and key module identification

**Fig. 2:**
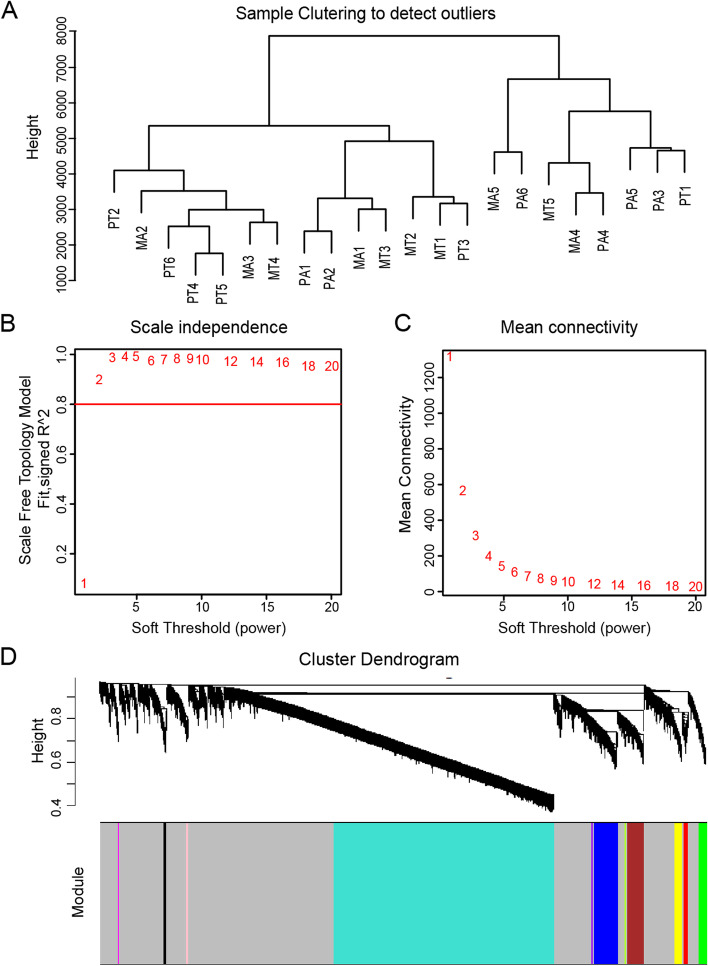
Construction of lncRNA co-expression clusters. **A** The sample clustering tree. **B** Variation of scale independence of co-expression networks under different soft thresholds. **C** Changes in mean connectivity of co-expression networks under different soft thresholds. **D** The hierarchical cluster dendrogram showing 12 co-expression lncRNA modules

**Fig. 3:**
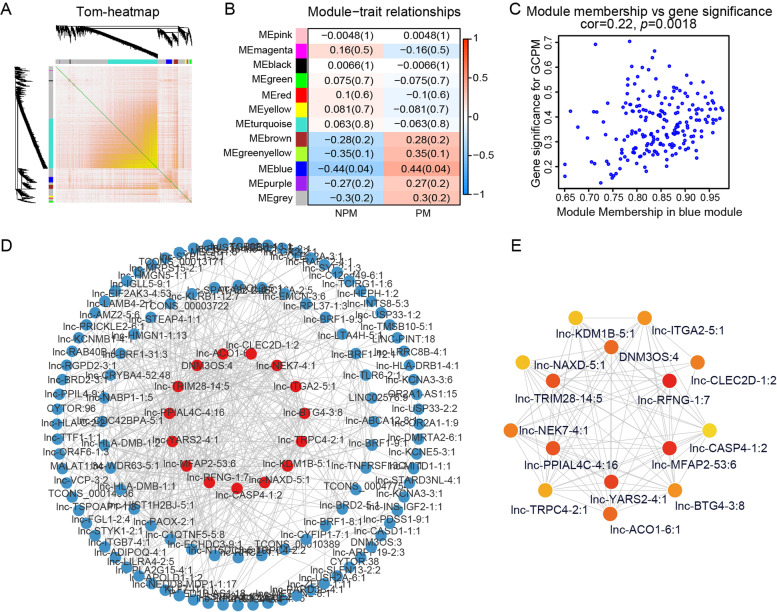
The correlations between lncRNA modules and GCPM. **A** Topological overlap matrix heatmap with one lncRNA per row and column. **B** Heatmap showing the lncRNA module-GCPM correlation. **C** Scatter plot showing lncRNA-trait significance (correlation between lncRNA expression and GCPM) and module membership (correlation between lncRNA expression and module eigengenes) in blue module. **D** The co-expression relationship between lncRNAs in blue modules was visualized using Cytoscape. **E** The top 15 lncRNAs with highest degree Calculated by cytoHubba

To evaluate specific lncRNAs that exert regulatory functions in gastric cancer peritoneal metastasis, we selected 5023 lncRNAs for subsequent WGCNA analysis. We first performed sample cluster analysis (Fig. 2A) and then applied the R package WGCNA. The power value was a vital parameter that could affect the independence and average connectivity degree of the co-expression modules. An approximate scale-free topology for the network was obtained using a soft-thresholding power of 3 (Fig. 2B, C). Therefore, a power value of 3 was selected for further analysis. As a result, a total of 12 modules marked with different colors were identified (Fig. 2D). The number of lncRNAs in the modules ranged from 10 to 2619. We subsequently constructed a TOM heatmap to visualize the co-expression relationships among all lncRNAs. Obviously, the co-expression relationship among lncRNAs within the same module was stronger than that among lncRNAs in different modules (Fig. 3A).

### lncRNA module-trait correlations with GCPM

To investigate the clinical significance of the module, we analyzed the correlation between clinical parameters and module eigengene. The results indicated that the blue module were positively associated with peritoneal metastasis (*r* = 0.44, *p* = 0.04) (Fig. 3B). Then, we made scatter plots showing module membership (MM) and gene significance (GS) for GCPM of all 199 lncRNAs in blue modules. A positive correlation was found between MM and GS (*r* = 0.22, *p* = 0.0018) (Fig. 3C, Table S3). We considered this correlation significant, although the correlation coefficient was not very high, and selected the blue module for subsequent analysis.

### GCPM-associated hub lncRNA identification and validation

**Fig. 4:**
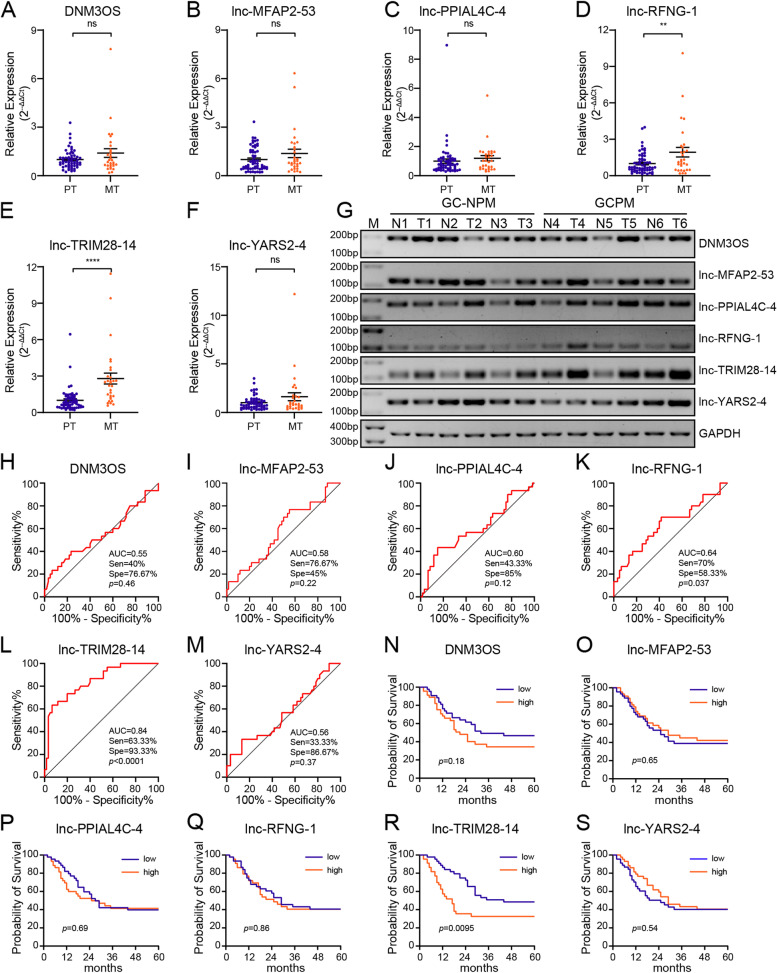
Tissue validation of candidate lncRNAs. **A**-**F** The expression levels of lncRNAs in gastric cancer tissue as determined by qRT-PCR. Student's t test, *, *p* < 0.05, **, *p* < 0.01, ***, *p* < 0.001, ****, *p* < 0.0001, ns, not significant. **G** Representative RT-PCR plots of the candidate lncRNAs. PCR products were separated by 2% agarose gel electrophoresis under 100 V for 1 h. Images were processed by Adobe Photoshop 22.1.1 software. GC-NPM, Gastric cancer patients without peritoneal metastasis; GCPM, Gastric cancer patients with peritoneal metastasis; N, tissue adjacent to tumor; T, tumor tissue. **H**-**M** ROC curve of six lncRNAs signature for the detection of GCPM. AUC, area under curve, Sen, sensitivity at the optimal cut-off value, Spe, specificity at the optimal cut-off value. **N**-**S** Kaplan-Meier survival analysis of overall survival (OS) for the lncRNA-high-expressed group and lncRNA-low-expressed group using log-rank test

We found that most lncRNAs in the blue module had high MM and GS. Therefore, it was extremely difficult to screen hub lncRNAs by limiting the threshold of these two parameters. With weighted correlation coefficient > 0.2 as the threshold, we found 199 nodes and 16,989 edges in the network constructed using lncRNAs from the blue module. Then, we selected and imported the top 500 edges into Cytoscape and obtained a large network system (Fig. 3D). We screened 15 candidate lncRNAs by calculating the degree with cytoHubba (Table S4). Finally, six lncRNAs with the top highest degree and MCC value (DNM3OS:4, lnc-MFAP2-53:6, lnc-PPIAL4C-4:16, lnc-RFNG-1:7, lnc-TRIM28-14:5, lnc-YARS2-4:1) were selected as candidate hub lncRNAs, henceforth referred to as DNM3OS, lnc-MFAP2-53, lnc-PPIAL4C-4, lnc-RFNG-1, lnc-TRIM28-14 and lnc-YARS2-4, respectively (Fig. 3E). Then, we performed qRT-PCR (Fig. 4A-F) and RT-PCR (Fig. 4G) using tumor tissues from 60 gastric cancer patients without peritoneal metastasis (PT) and 30 tumor tissues from gastric cancer patients with peritoneal metastasis (MT) to validate GCPM-related lncRNAs. The results showed that compared with PT, the expression of lnc-RFNG-1 (fold change 1.94, *p* = 0.0045) and lnc-TRIM28-14 (fold change 2.80, *p* < 0.0001) was significantly higher in MT. However, we found no evidence that the other four lncRNAs were also GCPM-related hub lncRNAs.

In addition, we drew the receiver operating characteristic (ROC) curve based on the expression of lnc-TRIM28-14 and lnc-RFNG-1 in the tumor tissues. AUC values for lnc-TRIM28-14 and lnc-RFNG-1 were 0.84 and 0.64, respectively. Moreover, the ROC curve inflection point indicated that lnc-TRIM28-14 had higher specificity (93.33%) but lower sensitivity (63.33%) than lnc-RFNG-1 (70% sensitivity and 58.33% specificity). However, DNM3OS, lnc-MFAP2-53, lnc-PPIAL4C-4, and lnc-YARS2-4 lacked the diagnostic efficiency of GCPM (Fig. 4H-M). Finally, the Kaplan-Meier survival analysis revealed the association between the expression of six lncRNAs and the overall survival of gastric cancer patients. We found that high expression of lnc-TRIM28-14 indicated a poor prognosis (*p* = 0.0095). Surprisingly, no association was found between the expression of lncRFNG-1, DNM3OS, lnc-MFAP2-53, lnc-PPIAL4C-4, and lnc-YARS2-4 and prognosis (*p* = 0.86, 0.18, 0.65, 0.69 and 0.54, respectively). (Fig. 4N-S). We further found that high expression of lnc-TRIM28-14 was positively correlated with poor tumor differentiation, peritoneal metastasis, and increased TNM stage (Table S7). Moreover, in a univariate Cox hazards analysis, tumor size, differentiation, depth of invasion and expression of lnc-TRIM28-14 were related to prognosis of the patients (Table S8).

### Gene co-expression network construction of mRNA and key module identification

**Fig. 5:**
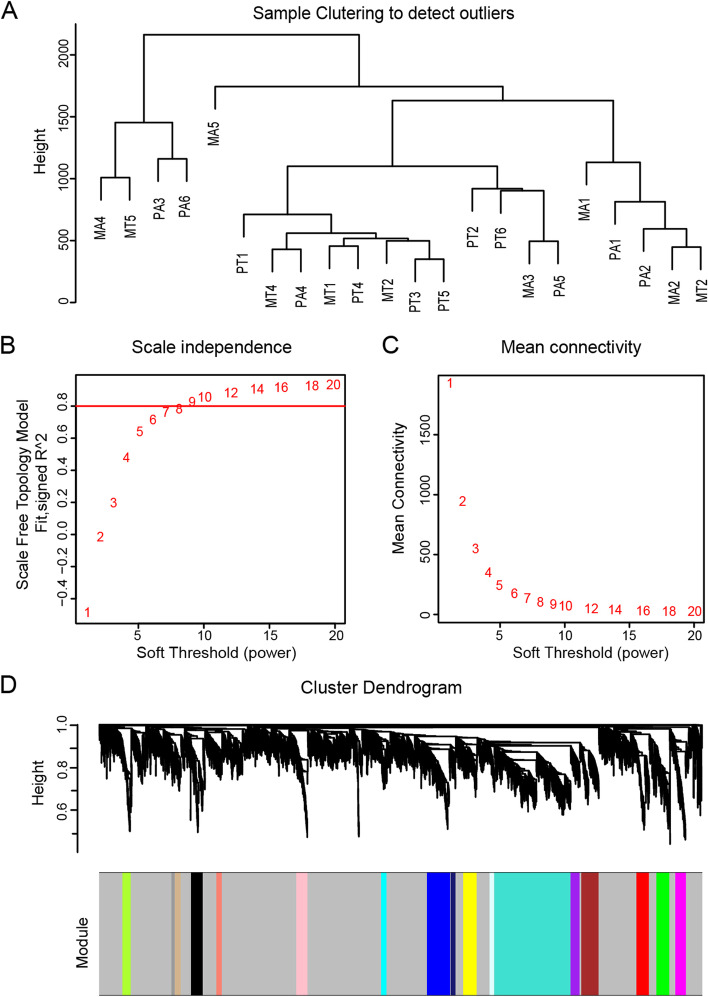
Construction of gene co-expression clusters. **A** The sample clustering tree. **B** Variation of scale independence of co-expression networks under different soft thresholds. **C** Changes in mean connectivity of co-expression networks under different soft thresholds. **D** The hierarchical cluster dendrogram shows 18 co-expression modules

**Fig. 6:**
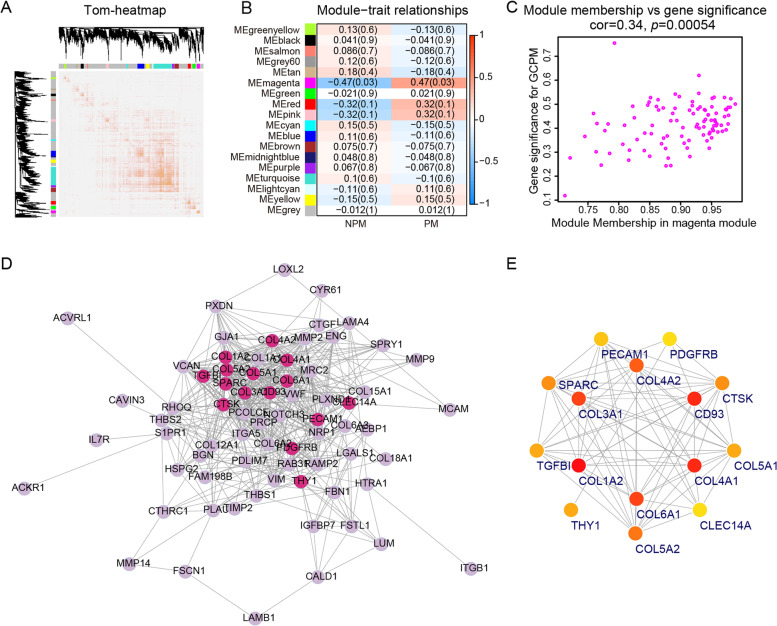
The correlation between gene modules and GCPM. **A** Topological overlap matrix heatmap with one gene per row and column. **B** Heatmap showing the correlation between gene modules and GCPM. **C** Scatter plot showing gene-trait significance (correlation between gene expression and GCPM) and module membership (correlation between gene expression and module eigengenes) in magenta module. **D** The co-expression relationship between genes in blue modules was visualized using Cytoscape. **E** Top 15 genes with highest degree calculated by cytoHubba

It was generally believed that lncRNAs regulated the function of genes by binding to miRNAs, proteins, etc., thereby affecting the occurrence and development of tumors. Therefore, we analyzed 5683 mRNAs from RNA-seq following the same approach. We first performed sample clustering analysis (Fig. 5A) and determined the power value as 10 by balancing scale independence and mean connectivity (Fig. 5B, C) and then divided all genes into 18 modules (Fig. 5D). The number of genes in the modules ranged from 30 to 3465. The TOM heatmap subsequently constructed showed that genes in the same module had a strong co-expression relationship (Fig. 6A).

### Module-trait correlation with GCPM and hub genes identification and validation

The magenta module was identified as associated with peritoneal metastases (*r* = 0.47, *p* = 0.03) (Fig. 6B). We visualized MM and GS of 100 genes of the magenta module, and found a significant correlation between them (*r* = 0.34, *p* = 0.00054 (Fig. 6C, Table S5). We constructed a gene co-expression network in Cytoscape with 500 top edges using a method similar to the one used above (Fig. 6D) and selected 15 genes with the highest degree and MCC value. Finally, six genes with highest degree (CD93, COL1A2, COL3A1, COL4A1, COL4A2 and COL6A1) were subsequently identified as candidate hub genes of GCPM (Fig. 6E, Table S6).

**Fig. 7:**
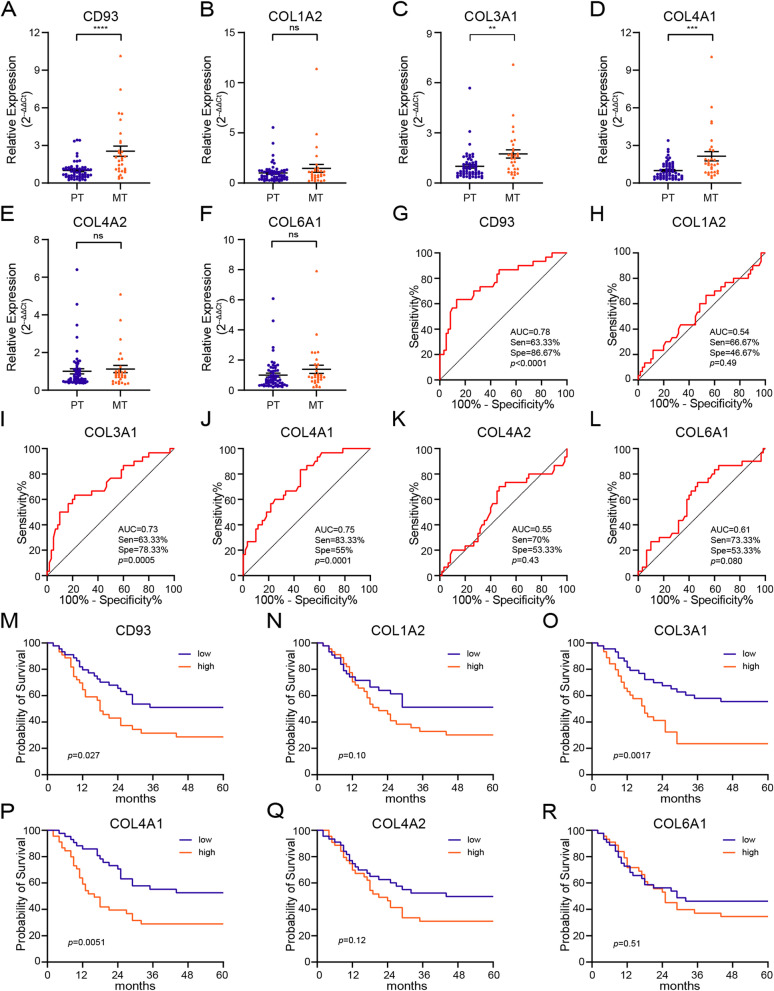
Tissue validation of candidate genes. **A**-**F** Expression level of candidate hub genes expression in gastric cancer tissue by qRT-PCR. Student's t test, *, *p* < 0.05, **, *p* < 0.01, ***, *p* < 0.001, ****, *p* < 0.0001, ns, not significant. **G**-**L** ROC curve of six genes signature used to determine GCPM. AUC, area under curve, Sen, sensitivity at the optimal cut-off value, Spe, specificity at the optimal cut-off value. **M**-**R** Kaplan-Meier analysis of the OS for the gene-high-expressed group and gene-low-expressed group using log-rank test

Then, we performed qRT-PCR with tumor tissues from 60 PT and 30 MT. The results showed that compared with PT, the expression of CD93 (fold change 2.54, *p* < 0.0001), COL3A1 (fold change 1.74, *p* = 0.0017) and COL4A1 (fold change 2.15, *p* = 0.0001) was significant higher in MT. However, the expression of COL1A2, COL4A2, and COL6A1 was not significantly different between the two groups (Fig. 7A-F). In addition, we drew the ROC curve based on the expression levels. The AUC values for CD93, COL3A1, and COL4A1 were 0.78, 0.73 and 0.75, respectively. ROC curve inflection point indicated that COL4A1 had the highest sensitivity (88.33%) followed by CD93 and COL3A1 both with 63.33% sensitivity. In contrast, CD93 showed the highest specificity of 86.67% followed by COL3A1 (78.33%) and COL4A1 ( 55%). We did not demonstrate the diagnostic efficacy of COL1A2, COL4A2, and COL6A1 for GCPM (Fig. 7G-L). Interestingly, we also anlysized peritoneal metastases from GCPM patients and found that CD93, COL3A1, COL4A1 expression were higher in metastases than in the primary tumor (Fig. S5).

Finally, the Kaplan-Meier survival analysis revealed the association between the three genes and the overall survival of gastric cancer patients. The high expression of CD93, COL3A1 and COL4A1 indicated their poor prognostic value (*p* = 0.027, 0.0017, 0.0051, respectively). But COL1A2, COL4A2, and COL6A1 were not significantly associated with prognosis (Fig. 7M-R). We further found that expression of CD93 was correlated with tumor size, differentiation and peritoneal metastasis; that of COL3A1 was correlated with differentiation, depth of invasion, peritoneal metastasis and TNM stage; that of COL4A1 was correlated with tumor size and peritoneal metastasis (Table S7). Moreover, expression of CD9, COL3A1, COL4A1 were related to prognosis of the patients through univariate Cox analysis and we finally determined that peritoneal metastasis, lnc-TRIM28-14 and COL3A1 expression could be independent risk factors for assessing the prognosis of patients with gastric cancer using multivariate Cox analysis (Table S8).

### GCPM-associated pathways and relationship between GCPM-related lncRNAs and genes

**Fig. 8:**
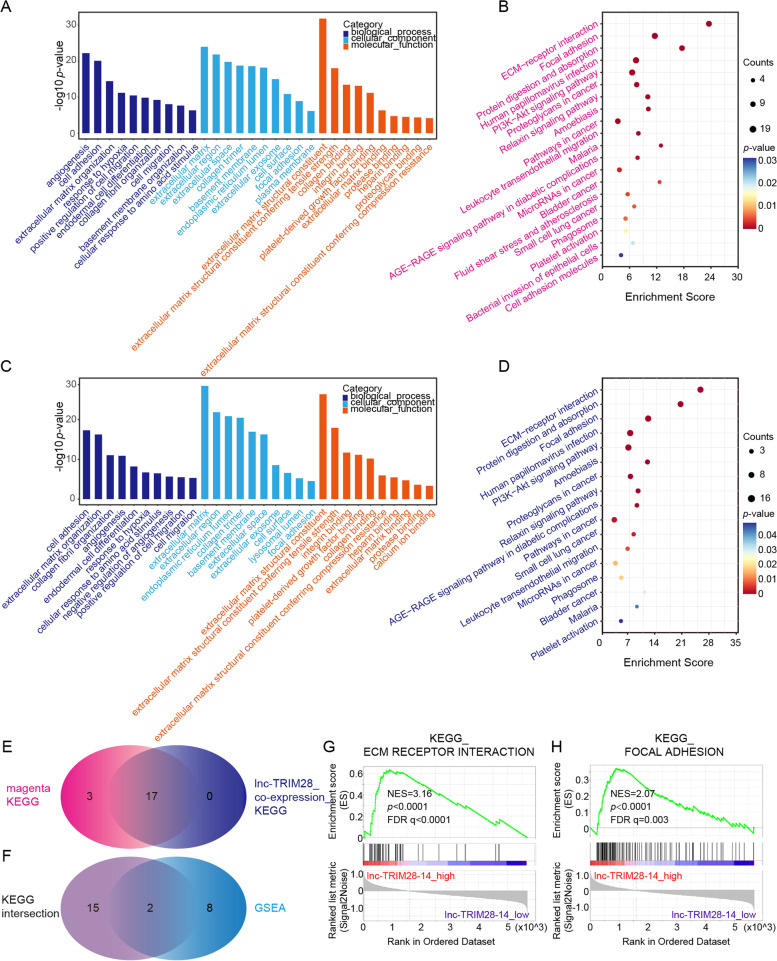
GCPM-related pathway analysis. **A**-**B**. GO terms and KEGG pathways enriched by megenta module genes. **C**-**D** GO terms and KEGG pathways enriched by lnc-TRIM28-14 co-expressed genes. **E** Venn plot for KEGG terms between B and D. **F** Venn plot for pathways associated with the expression of lnc-TRIM28-14 by GSEA and overlapping pathways in E. **G**-**H** GSEA analysis showed that high expression of lnc-TRIM-28–14 was significantly associated with ECM-receptor interaction and focal adhesion

We considered how GCPM-related genes mediated peritoneal metastasis. Therefore, we performed GO and KEGG pathway analyses for the genes in magenta module. Interestingly, the 100 genes were enriched in extracellular matrix and associated with multiple pathways including ECM-receptor interaction, focal adhesion and PI3K-AKT signaling pathway (Fig. 8A, B).

We considered how hub lncRNAs mediated peritoneal metastasis. Therefore, we performed correlation analysis with RNA-seq data. We found 101 genes co-expressed with lnc-TRIM28-14 and 10 genes co-expressed with lnc-RFNG with correlation coefficient > 0.6, *p* < 0.05 as the threshold (Fig. S2A). Interestingly, 65 of these 101 genes co-expressed with lnc-TRIM28-14 overlapped with the 100 genes in the magenta module (Fig. S2B), and 6 candidate hub genes, such as CD93, were included in the intersection (Fig. S2C). And the PCA plot also showed lnc-TRIM28-14 had potential co-expression relationship with hub genes (Fig. S2D). Subsequent GO and KEGG analyses suggested that these 101 genes were also enriched in similar pathways (Fig. 8C, D). Taking the intersection of two KEGG results, we obtained 17 overlapping pathways, including ECM-receptor interaction, focal adhesion and PI3K-AKT signaling pathway (Fig. 8E). Subsequently, to find the key pathway of GCPM, we performed GSEA, and we found that the high expression of lnc-TRIM28-14 was related to 10 KEGG pathways, including ECM-receptor interaction and focal adhesion (Fig. 8F-H, S3).

**Fig. 9:**
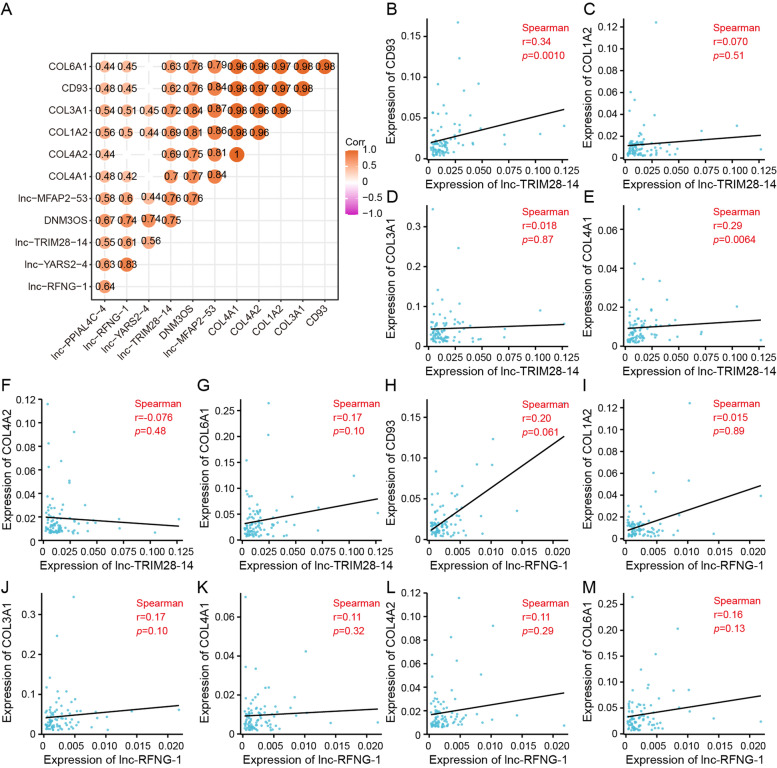
Identification of the relationship between hub lncRNAs and genes. **A** Correlation heatmap of lncRNAs and genes determined by RNA-seq with the threshold of *p* < 0.05. **B**-**G** Expression correlation analysis for lnc-TRIM28-14 and 6 candidate hub genes determined by qRT-PCR (normalized to GAPDH). **H**-**M** Expression correlation analysis for lnc-RFNG-1 and 6 candidate hub genes based on qRT-PCR assay (normalized to GAPDH)

Based on this analysis, we considered that GCPM-related lncRNAs and genes may have potential associations. Therefore, we performed a visual analysis of the correlation between 6 candidate hub lncRNAs and 6 candidate hub genes using our RNA-seq data and found that lnc-TRIM28-14 was strongly correlated with 6 candidate hub genes whereas lnc-RFNG-1 was weakly correlated with these genes (Fig. 9A). Finally, we used tissue qRT-PCR results for verification and found that expression of lnc-TRIM28-14 was positively correlated with that of CD93 (*r* = 0.34, *p* = 0.0010) and COL4A1 (*r* = 0.29, *p* = 0.0064) (Fig. 9B-G). Consistent with the RNA-sequencing data, there was no evidence that tissue-level lnc-RFNG-1 was associated with these six genes (Fig. 9H-M).

## Discussion

Peritoneal metastasis is a common metastasis mode and an end state of gastric cancer usually associated with poor prognosis. Based on the seed-soil theory, gastric cancer peritoneal metastasis is a polymorphic and multi-molecular regulated process, including changes in seeds and soil and the regulation of multiple RNAs [15, 16]. Many scholars have made efforts to explore the molecular mechanism underlying GCPM and find new methods for the diagnosis and treatment of GCPM. LIMK1 was reported to mediate GCPM [17], whereas miR-30a-5p, -659-3p, and -3917 were reported to be potential biomarkers of GCPM [18]. Recent studies have found several lncRNAs with important roles in GCPM [19–21].

However, no research has been conducted on GCPM-related lncRNA based on WGCNA, which is a more systematic biological algorithm. We performed RNA sequencing, constructed a co-expression network with as many lncRNAs as possible using WGCNA and found GCPM-related blue module and identified six candidate hub lncRNAs (DNM3OS, lnc-MFAP2-53, lnc-PPIAL4C-4, lnc-RFNG-1, lnc-TRIM28-14, lnc-YARS2-4) associated with GCPM. Subsequently, we performed tissue qRT-PCR, RT-PCR validation and ROC curve, which revealed that the expression of lnc-TRIM28-14 and lnc-RFNG-1 was associated with GCPM. Moreover, high expression of lnc-TRIM28-14 indicated a poor prognosis. However, we did not demonstrate that expression of lnc-RFNG-1 was associated with prognosis, probably due to the low number of cases used, individual differences and the proportion of patients with and without peritoneal metastasis in our cases.

The lncRNA lnc-TRIM28-14 is an intergenic sequence transcribed from chromosome 19. It occurs adjacent to tripartite motif-containing protein 28 (TRIM28) gene. The TRIM28 gene belonged to Transcriptional Intermediary Factor 1 family and worked as one of key regulators of development and differentiation [22]. In recent years, studies have found that TRIM28 could regulate tumor progression and promote tumor growth by enhancing TRIM24 stability [23]. Another study found that TRIM28 inhibited immunostimulatory cytokine expression [24]. A recent study found that lncRNA could bind to TRIM28 to inhibit its function [25]. Interestingly, TRIM28 was reported to be associated with GCPM [26]. Therefore, it was worth considering whether there was a potential association between lnc-TRIM28-14 and TRIM28 in expression or function. Lnc-RFNG-1 is an intronic lncRNA derived from Ofucosylpeptide 3-beta-N-acetylglucosaminyltransferase (RFNG) gene. RFNG, a member of Fringe proteins, has been reported to regulate cell growth and differentiation in a NOTCH-dependent manner [27–29]. To date, little research has been done on RFNG, and even less is known about its role in tumors. Only one study reported that RFNG might be involved in the progression of hepatocellular carcinoma [30]. Therefore, Lnc-RFNG-1 opens up new insights into how genes work independently of proteins.

lncRNAs are often considered to play a role in regulating gene expression. Therefore, we also performed WGCNA on mRNA sequencing data. We found GCPM-related magenta modules and screened six candidate hub genes (CD93, COL1A2, COL3A1, COL4A1, COL4A2, and COL6A1). GCPM-related CD93, COL3A1 and COL4A1 were identified using tissue qRT-PCR and ROC curve analysis. Kaplan–Meier survival analysis suggested that high expression of CD93, COL3A1 and COL4A1 indicated a poor prognosis. Unfortunately, we did not verify that the expression of COL1A2, COL4A2, and COL6A1 was associated with GCPM and prognosis, most likely due to our limited sample size. CD93 is a transmembrane protein, which has received increasing research attention in recent years. Studies found that CD93 promoted endothelial cells β1 integrin activation during tumor angiogenesis [31] and blockade of CD93 can promote tumor vascular maturation, improving drug delivery and immune microenvironment [32]. COL3A1 and COL4A1 are important components of the extracellular matrix. COL3A1 was reported to promote renal cell carcinoma growth and metastasis [33] whereas COL4A1 facilitated hepatocellular carcinoma cells proliferation, migration and invasion by activating FAK-Src signaling [34]. Bioinformatics analysis suggested that H19 promoted gastric carcinogenesis by promoting COL3A1 and COL4A1 expression [35]. However, the association of these molecules with GCPM has not been reported.

We considered how ln-TRIM28-14 and lnc-RFNG-1 mediated GCPM. Subsequent GO and KEGG analyses suggested that hub lncRNAs and genes were enriched in similar pathways. Using GSEA, we identified ECM-receptor interaction and focal adhesion as the potential key pathways of GCPM. Therefore, we speculated that the “seed” gastric cancer cells enhanced invasiveness by interacting with the matrix, thus mediating peritoneal metastasis. We considered whether there was an expression regulation relationship between hub lncRNAs and genes. Consequently, we analyzed the qRT-PCR results. Finally, we verified that expression of lnc-TRIM28-14 was positively correlated with that of CD93 and COL4A1. In recent years, studies found that lncRNAs can regulate gene expression and present various patterns [36], such as competing-endogenous-RNAs pattern [37], transcription regulation [38] and alternative splicing [39].

Unfortunately, due to time constraints, we did not conduct more experiments to verify the regulatory relationship of lnc-TRIM28-14 with CD93 and COL4A1. We considered to verify the specific roles and molecular mechanisms of these lncRNAs and genes mediating GCPM in our follow-up study. In addition, we found no association between the expression of ln-RFNG-1 and that of hub genes. We speculated that ln-RFNG-1 might regulate the biological behavior of tumor cells in an unconventional pattern, promoting seed invasion and colonization into the abdominal cavity.

## Conclusion

In this study, WGCNA was performed using RNA-seq data from tissue samples from patients with and without peritoneal metastases. GCPM-related lncRNAs (lnc-TRIM28-14, lnc-RFNG-1) and genes (CD93, COL3A1, and COL4A1) were identified, as well as key pathways that might mediate GCPM. Moreover, we found that lnc-TRIM28-14 has potential regulatory relationship with CD93 and COL4A1 through correlation analysis, although more verification experiments are needed. These molecules could provide promising new avenues for gastric cancer diagnosis and treatment.

## Supplementary Information

**Additional file 1** 

**Additional file 2:** **Table S2.** Primers of lncRNAs and genes for RT-PCR and qRT-PCR.

**Additional file 3:** **Table S3.** LncRNAs in blue module.

**Additional file 4:** **Table S4.** The top 15 lncRNAs ranked by degree in blue module.

**Additional file 5:** **Table S5.** Genes in magenta module.

**Additional file 6:** **Table S6.** The top 15 genes ranked by degree in magenta m

**Additional file 7:** **Table S7.** The correlation between clinicopathological parameters and expression of hub lncRNAs and genes in 90 GC patients.

**Additional file 8:** **Table S8.** Univariate and multivariate Cox analyses of various potential prognostic factors in GC patients.

**Additional file 9:** **Figure S1.** Functional analysis of DElncRNAs. (A, B) GO and KEGG analysis of DElncRNAs in PTvsPA. (C, D) GO and KEGG analysis of DElncRNAs in MTvsMA. (E, F) GO and KEGG analysis of DElncRNAs in MTvsPT. The DElncRNAs enriched GO terms and KEGG pathways were ranked according to -log10(*p*-value) or *p*-value, and the top 10 GO terms and top 20 KEGG pathways were presented.

**Additional file 10:** **Figure S2.** Correlation analysis of hub lncRNAs and genes. (A) The lncRNA-mRNA correlation network. Based on the correlation coefficient > 0.6, *p* < 0.05 as the cut-off values, the closer the distance is, the higher the correlation coefficient. (B) Venn analysis for co-expressed genes of lnc TRIM28-14 and genes in magenta module. (C) Venn analysis showing that 6 candidate genes exactly included in the intersection in B. (D) PCA plot showing the correlation between GCPM related biomarkers.

**Additional file 11:** **Figure S3.** GSEA analysis of pathways associated with the expression of lnc-TRIM28-14. (A H) The remaining eight KEGG pathways significantly associated with the expression of lnc TRIM28-14 (*p*<0.05, FDR q<0.2.

**Additional file 12:** **Figure S4.** Raw agarose gel electrophoresis images for hub lncRNAs analysis.(A-G) The PCR products were analyzed with agarose gel electrophoresis on a 2% agarose gel, and expression of six hub lncRNAs and GAPDH was shown. The 12 lanes were N1, T1, N2, T2, N3, T3, N4, T4, N5, T5, N6, T6. The first 6 samples were derived from paracancerous (N) and cancerous (T) tissues from 3 gastric cancer patients without peritoneal metastasis, and the last 6 samples were derived from paracancerous (N) and cancerous (T) tissues from 3 gastric cancer patients with peritoneal metastases.

**Additional file 13:** **Figure S5.** Further verification of GCPM-related genes. (A-C) CD93, COL3A1 and COL4A1 expression in different gastric cancer tissues determined by qRT-PCR. (PT, primary tumors without GCPM; MT, primary tumors with GCPM; PM, GC peritoneal metastases)Student's t test, *, *p* < 0.05, **, *p* < 0.01, ***, *p* < 0.001, ****, *p* < 0.0001, ns, not significant.

## Data Availability

All RNA sequencing data of this study are available in Science Data Bank (ScienceDB, https://www.scidb.cn/en) by visiting http://doi.org/10.57760/sciencedb.03133, and the processed lncRNA data is available in the supplementary file (Table S1). The other resources used in this study are available from the corresponding authors upon reasonable request.

## Abbreviations

GCPM: Gastric Cancer Peritoneal Metastasis
lncRNA: Long non-coding RNA
RNA-seq: RNA sequencing
GO: Gene Ontology
KEGG: Kyoto Encyclopedia of Genes and Genomes
WGCNA: Weighted Gene Co-expression Network Analysis
ROC curve: Receiver Operating Characteristic curve
AUC: Area Under Curve
qRT-PCR: Quantitative real-time PCR
GSEA: Gene Set Enrichment Analysis
GC: Gastric Cancer
PT: Non-metastatic gastric cancer primary tumor
PA: Normal gastric mucosal tissue adjacent to PT
MT: Gastric cancer primary tumor with peritoneal metastasis
MA: Normal gastric mucosal tissue adjacent to MT
DElncRNAs: Differentially expressed lncRNAs
BP: Biological process
CC: Cellular component
MF: Molecular function
GS: Gene significance
MM: Module membership
