# Supplementary material for: Identification and validation of crucial lnc-TRIM28-14 and hub genes promoting gastric cancer peritoneal metastasis
Source: BMC Cancer. 2023 Jan 23;23:76. doi: 10.1186/s12885-023-10544-8 (PMC9872371; doi:10.1186/s12885-023-10544-8)
Supplement: Supplementary file 2 — Additional file 2: Table S2. Primers of lncRNAs and genes for RT-PCR and qRT-PCR. [file 12885_2023_10544_MOESM2_ESM.pdf]

Table S2. Primers of lncRNAs and genes for RT-PCR and qRT-PCR

Primer Name	Length of Product(bp)	Sequence, 5'-3'
DNM3OS-FP	192	CCCCTGGATGTAATAAGTGTTGC
DNM3OS-RP		GTGTTATTGTTGCTGCATTCAGTG
lnc-MFAP2-53-FP	122	TTAGAGCGCAGAATGGGGAA
lnc-MFAP2-53-RP		AGTTCCCAGACGCCTAGTTT
lnc-PPIAL4C-4-FP	172	GAAAGCTGAAAGCGTGCGAG
lnc-PPIAL4C-4-RP		AGTCTGCACAGTTGTCCTGC
lnc-RFNG-1-FP	102	TCCATGGAGGGAAATGGGAC
lnc-RFNG-1-RP		GGAGCCACGTGAAGTAAGA
lnc-TRIM28-14-FP	101	GGTGCTCGCACAAGAAAGC
lnc-TRIM28-14-RP		CCTGGAAGAGGCTCATGGTT
lnc-YARS2-4-FP	154	AACTTCCAGTATGACCACACCC
lnc-YARS2-4-RP		CAGGCTAACTTCTGAAAAATCCTGT
CD93-FP	192	TGGAGAACCAGTACAGTCCGA
CD93-RP		TCCAAGGGGCCTTTAAGGAG
COL1A2-FP	141	CAGCCGGAGATAGAGGACCA
COL1A2-RP		ACTGAGCAGCAAAGTTCCCA
COL3A1-FP	212	TGAAAGGACACAGAGGCTTCG
COL3A1-RP		TGGTTGACCATCACTGCCTC
COL4A1-FP	163	GGGGAGCCTGGTGAGTTTTA
COL4A1-RP		TCAATCCTACAGAACCCGGC
COL4A2-FP	117	CAGCATGGGGAGAGACCAG
COL4A2-RP		AACTTCTTCACACCCGCCAA
COL6A1-FP	158	TCTGAGCATCATCGCCACG
COL6A1-RP		ATTCGAAGGAGCAGCACACT
GAPGH-FP	345	TCGGAGTCAACGGATTTGGT
GAPDH-RP		TGATGACCCTTTTGGCTCCC

FP: Forward Primer; RP: Reverse Primer
