# Supplementary material for: Identification and validation of crucial lnc-TRIM28-14 and hub genes promoting gastric cancer peritoneal metastasis
Source: BMC Cancer. 2023 Jan 23;23:76. doi: 10.1186/s12885-023-10544-8 (PMC9872371; doi:10.1186/s12885-023-10544-8)
Supplement: Supplementary file 4 — Additional file 4: Table S4. The top 15 lncRNAs ranked by degree in blue module. [file 12885_2023_10544_MOESM4_ESM.pdf]

Table S4. The top 15 lncRNAs ranked by degree in blue module

node_name	Degree	MCC
lnc-RFNG-1:7	28	1662
lnc-MFAP2-53:6	25	4374
lnc-YARS2-4:1	24	6740
lnc-TRIM28-14:5	22	1580
lnc-PPIAL4C-4:16	22	4107
DNM3OS:4	21	1886
lnc-ACO1-6:1	21	184
lnc-NEK7-4:1	20	281
lnc-CLEC2D-1:2	20	138
lnc-ITGA2-5:1	19	536
lnc-BTG4-3:8	19	346
lnc-TRPC4-2:1	18	632
lnc-NAXD-5:1	16	525
lnc-KDM1B-5:1	16	735
lnc-CASP4-1:2	15	234

gree of lncRNAs was calculated by cytoHubba plugin 0.1 (http://apps.cytoscape.org/apps/cytohubl
