# Supplementary material for: Identification and validation of crucial lnc-TRIM28-14 and hub genes promoting gastric cancer peritoneal metastasis
Source: BMC Cancer. 2023 Jan 23;23:76. doi: 10.1186/s12885-023-10544-8 (PMC9872371; doi:10.1186/s12885-023-10544-8)
Supplement: Supplementary file 6 — Additional file 6: Table S6. The top 15 genes ranked by degree in magenta m [file 12885_2023_10544_MOESM6_ESM.pdf]

Table S6. The top 15 genes ranked by degree in magenta m

node_name	Degree	MCC
COL1A2	28	8452
CD93	22	763
COL4A1	22	8604
COL6A1	20	7064
COL3A1	20	3842
COL4A2	19	7638
COL5A2	18	3951
CTSK	17	754
SPARC	17	786
THY1	16	116
TGFBI	16	152
COL5A1	16	712
PECAM1	15	338
CLEC14A	14	576
PDGFRB	14	472

Degree of genes was calculated by cytoHubba plugin 0.1 (http://apps.
