# Supplementary material for: Identification and validation of crucial lnc-TRIM28-14 and hub genes promoting gastric cancer peritoneal metastasis
Source: BMC Cancer. 2023 Jan 23;23:76. doi: 10.1186/s12885-023-10544-8 (PMC9872371; doi:10.1186/s12885-023-10544-8)
Supplement: Supplementary file 7 — Additional file 7: Table S7. The correlation between clinicopathological parameters and expression of hub lncRNAs and genes in 90 GC patients. [file 12885_2023_10544_MOESM7_ESM.pdf]

Table S7. The correlation between clinicopathological parameters and expression of hub lncRNAs and genes in 90 GC patients.

Parameters	Cases	lnc-TRIM28-14 expression			CD93 expression			COL3A1 expression			COL4A1 expression		
		Low	High	*p* value	Low	High	*p* value	Low	High	*p* value	Low	High	*p* value
Age (years)													
<60	39	22	17	0.288	21	18	0.523	19	20	0.832	24	15	0.056
⩾60	51	23	28		24	27		26	25		21	30	
Gender													
Female	25	10	15	0.239	11	14	0.480	12	13	0.814	9	16	0.099
Male	65	35	30		34	31		33	32		36	29	
Tumor size (cm)													
<5	35	20	15	0.280	24	11	0.005	21	14	0.130	23	12	0.017
⩾5	55	25	30		21	34		24	31		22	33	
Differentiation													
Moderately-well	31	23	8	0.001	20	11	0.046	21	10	0.015	19	12	0.120
Poorly	59	22	37		25	34		24	35		26	33	
Depth of invasion													
T1+T2	32	17	15	0.660	20	12	0.078	23	9	0.002	19	13	0.186
T3+T4	58	28	30		25	33		22	36		26	32	
Lymph node invasion													
No	19	13	6	0.071	9	10	0.796	7	12	0.197	8	11	0.438
Yes	71	32	39		36	35		38	33		37	34	
Peritoneal metastasis													
No	60	36	24	0.007	37	23	0.002	39	21	<0.001	35	25	0.025
Yes	30	9	21		8	22		6	24		10	20	

TNM stage													
I + II	29	19	10	0.042	18	11	0.114	24	5	<0.001	17	12	0.259
III + IV	61	26	35		27	34		21	40		28	33	
