# Supplementary material for: Identification and validation of crucial lnc-TRIM28-14 and hub genes promoting gastric cancer peritoneal metastasis
Source: BMC Cancer. 2023 Jan 23;23:76. doi: 10.1186/s12885-023-10544-8 (PMC9872371; doi:10.1186/s12885-023-10544-8)
Supplement: Supplementary file 8 — Additional file 8: Table S8. Univariate and multivariate Cox analyses of various potential prognostic factors in GC patients. [file 12885_2023_10544_MOESM8_ESM.pdf]

Table S8. Univariate and multivariate Cox analyses of various potential prognostic factors in GC patients

	Univariate Cox analysis		Multivariate Cox analysis	
	HR (95%CI)	*p* value	HR (95%CI)	*p* value
Age (≥60/<60)	1.619(0.898-2.916)	0.109	-	-
Gender(male/female)	0.648(0.356-1.182)	0.157	-	-
Tumor size (≥5cm/<5cm)	1.91(1.048-3.479)	0.035	1.400(0.691-2.838)	0.351
Differentiation (poorly/ moderately-well)	2.068(1.108-3.860)	0.022	1.231(0.616-2.460)	0.557
Depth of invasion (T3+T4/T1+T2)	2.073(1.109-3.915)	0.025	1.432(0.707-2.898)	0.318
Lymph node invasion (Yes/No)	1.05(0.537-2.056)	0.886	-	-
Peritoneal metastasis (Yes/No)	3.036(1.703-5.412)	<0.001	2.283(1.237-4.212)	0.008
TNM Stage (III-IV/I-II)	2.692(1.367-5.300)	0.004	1.594(0.715-3.554)	0.254
lnc-TRIM28-14 expression (high/low)	2.071(1.172-3.660)	0.012	1.971(1.077-3.608)	0.028
CD93 expression (high/low)	1.87(1.058-3.305)	0.031	1.164(0.593-2.285)	0.658
COL3A1 expression (high/low)	2.443(1.364-4.376)	0.003	1.268(0.587-2.741)	0.546
COL4A1 expression (high/low)	2.212(1.241-3.944)	0.007	2.206(1.211-4.016)	0.01

HR: hazard ratio; CI: confidence interval.
