# Supplementary material for: Identification and validation of crucial lnc-TRIM28-14 and hub genes promoting gastric cancer peritoneal metastasis
Source: BMC Cancer. 2023 Jan 23;23:76. doi: 10.1186/s12885-023-10544-8 (PMC9872371; doi:10.1186/s12885-023-10544-8)
Supplement: Supplementary file 9 — Additional file 9: Figure S1. Functional analysis of DElncRNAs. (A, B) GO and KEGG analysis of DElncRNAs in PTvsPA. (C, D) GO and KEGG analysis of DElncRNAs in MTvsMA. (E, F) GO and KEGG analysis of DElncRNAs in MTvsPT. The DElncRNAs enriched GO terms and KEGG pathways were ranked according to -log10(p-value) or p-value, and the top 10 GO terms and top 20 KEGG pathways were presented. [file 12885_2023_10544_MOESM9_ESM.pdf]

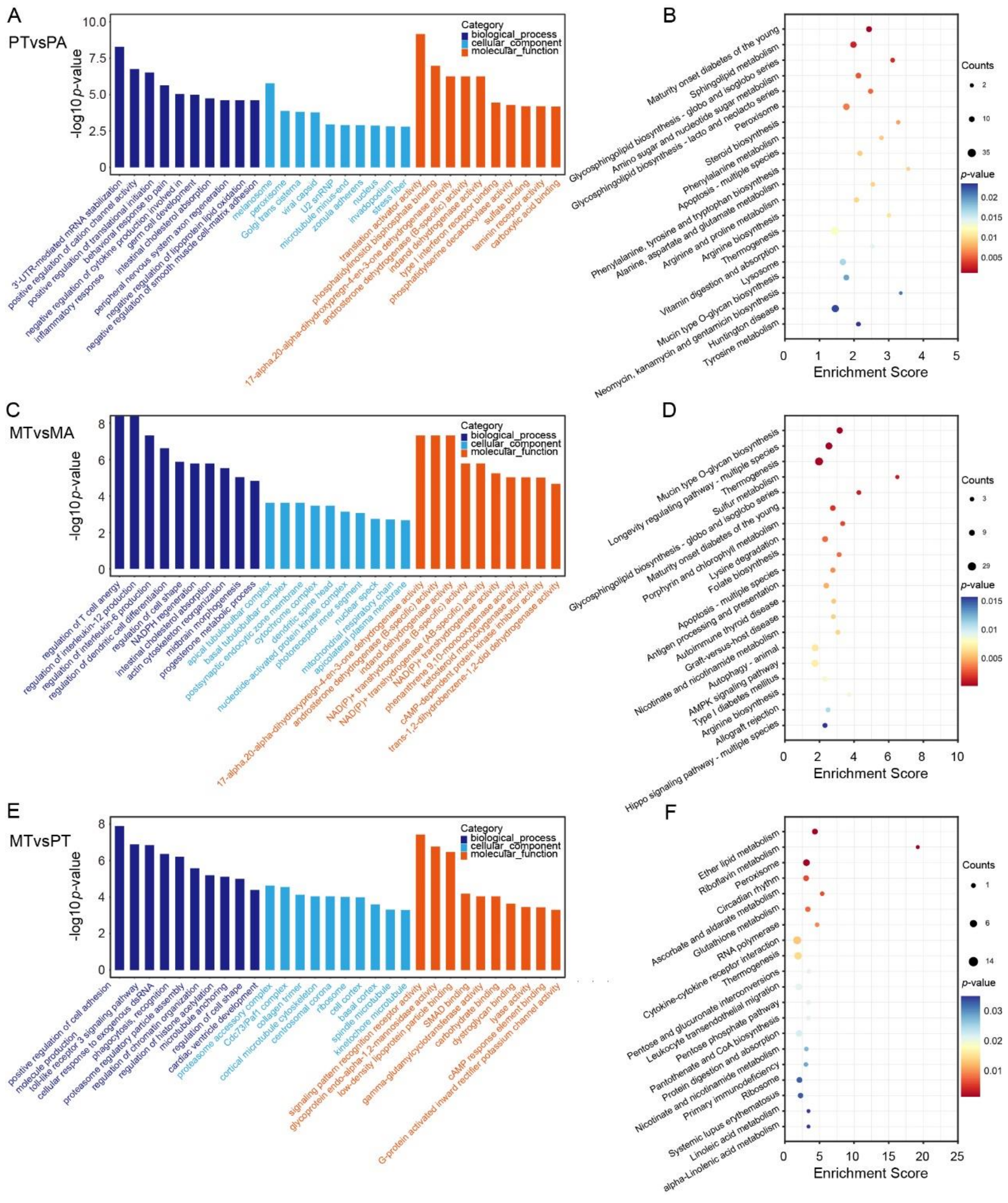

Figure S1 Functional analysis of DElncRNAs. (A, B) GO and KEGG analysis of DElncRNAs in PTvsPA. **(C, D)** GO and KEGG analysis of DElncRNAs in MTvsMA. **(E, F)** GO and KEGG analysis of DElncRNAs in MTvsPT. The DElncRNAs enriched GO terms and KEGG pathways were ranked according to -log10(*p*-value) or *p*-value, and the top 10 GO terms and top 20 KEGG pathways were presented.
