# Supplementary material for: Identification and validation of crucial lnc-TRIM28-14 and hub genes promoting gastric cancer peritoneal metastasis
Source: BMC Cancer. 2023 Jan 23;23:76. doi: 10.1186/s12885-023-10544-8 (PMC9872371; doi:10.1186/s12885-023-10544-8)
Supplement: Supplementary file 11 — Additional file 11: Figure S3. GSEA analysis of pathways associated with the expression of lnc-TRIM28-14. (A H) The remaining eight KEGG pathways significantly associated with the expression of lnc TRIM28-14 (p<0.05, FDR q<0.2. [file 12885_2023_10544_MOESM11_ESM.pdf]

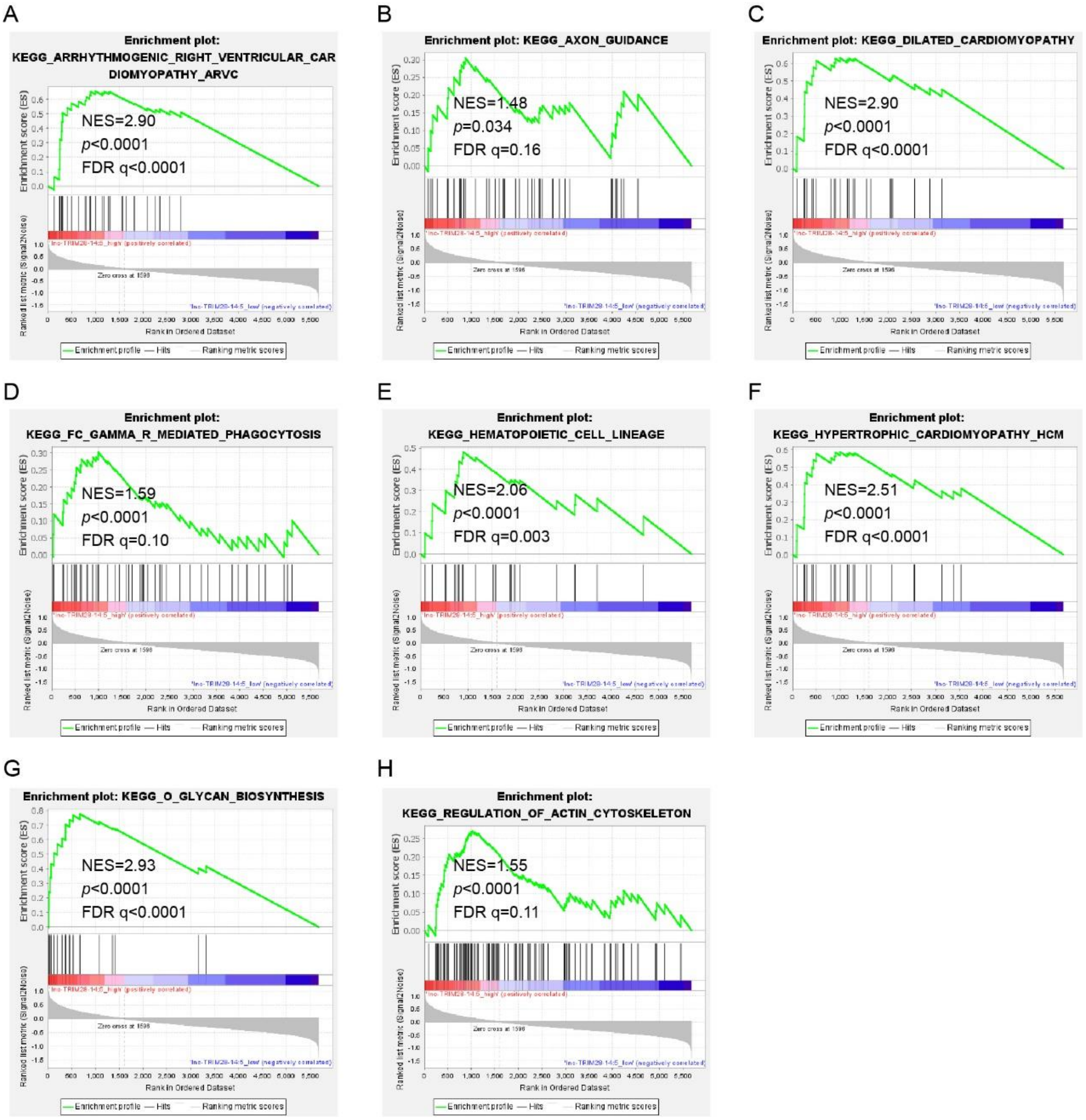

Figure S3 GSEA analysis of pathways associated with the expression of lnc-TRIM28-14. (A-H) The remaining eight KEGG pathways significantly associated with the expression of lnc-TRIM28-14 (p<0.05, FDR q<0.25).
