# Supplementary material for: Identification and validation of crucial lnc-TRIM28-14 and hub genes promoting gastric cancer peritoneal metastasis
Source: BMC Cancer. 2023 Jan 23;23:76. doi: 10.1186/s12885-023-10544-8 (PMC9872371; doi:10.1186/s12885-023-10544-8)
Supplement: Supplementary file 12 — Additional file 12: Figure S4. Raw agarose gel electrophoresis images for hub lncRNAs analysis.(A-G) The PCR products were analyzed with agarose gel electrophoresis on a 2% agarose gel, and expression of six hub lncRNAs and GAPDH was shown. The 12 lanes were N1, T1, N2, T2, N3, T3, N4, T4, N5, T5, N6, T6. The first 6 samples were derived from paracancerous (N) and cancerous (T) tissues from 3 gastric cancer patients without peritoneal metastasis, and the last 6 samples were derived from paracancerous (N) and cancerous (T) tissues from 3 gastric cancer patients with peritoneal metastases. [file 12885_2023_10544_MOESM12_ESM.pdf]

A

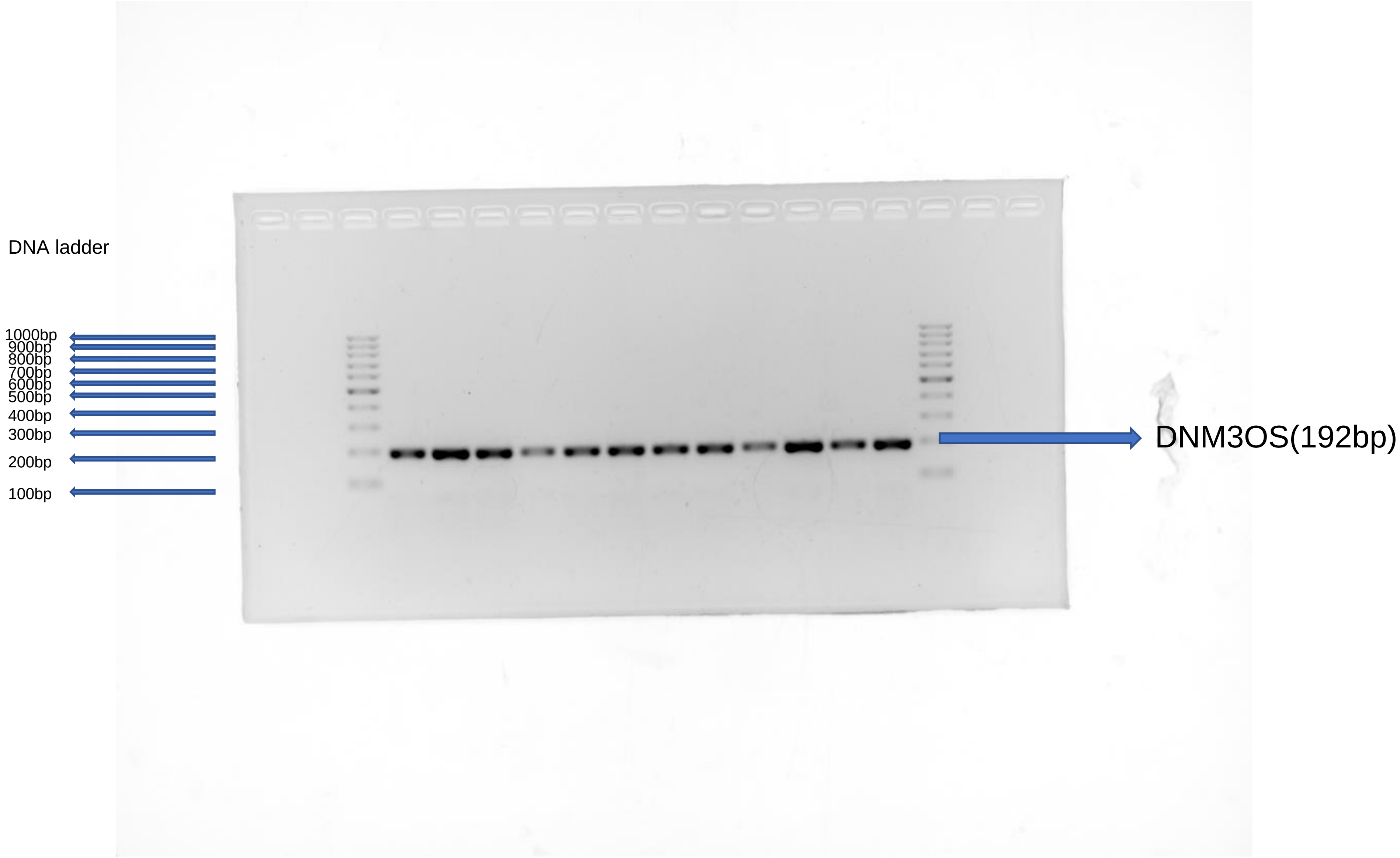

DNA ladder
1000bp
900bp
800bp
700bp
600bp
500bp
400bp
300bp
200bp
100bp
DNM3OS(192bp)

B

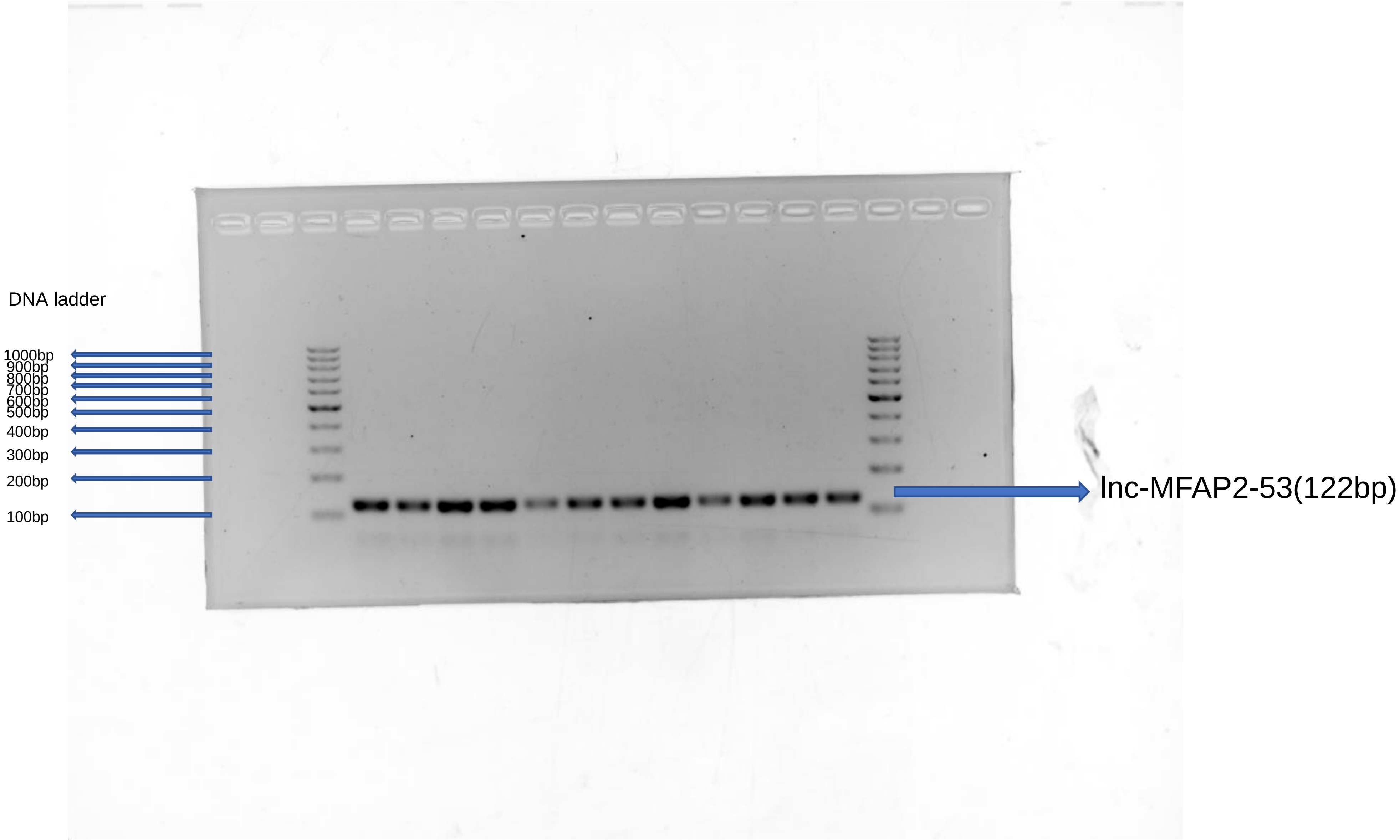
DNA ladder
1000bp
900bp
800bp
700bp
600bp
500bp
400bp
300bp
200bp
100bp
lnc-MFAP2-53(122bp)

C

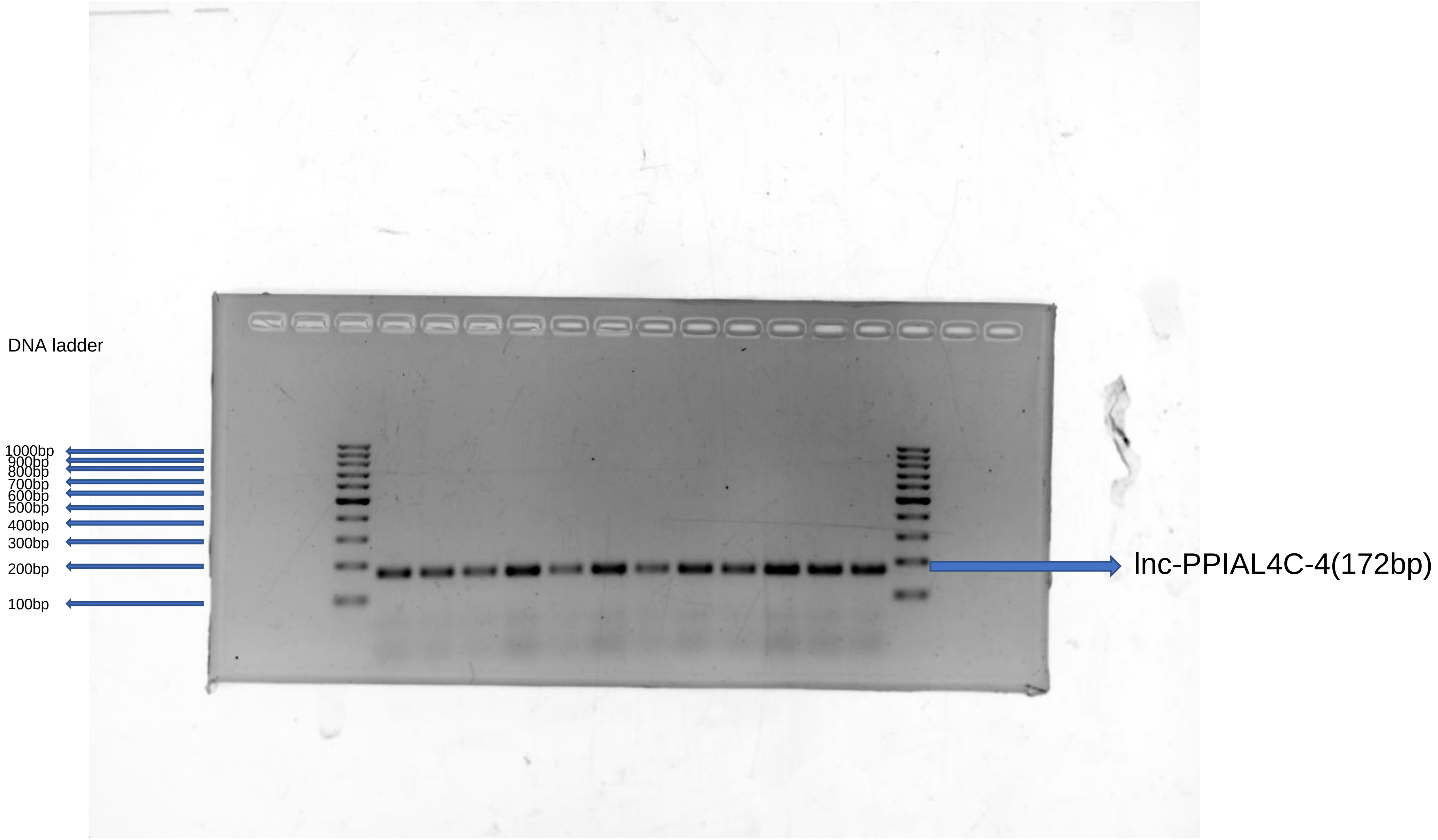
DNA ladder
1000bp
900bp
800bp
700bp
600bp
500bp
400bp
300bp
200bp
100bp
lnc-PPIAL4C-4(172bp)

D

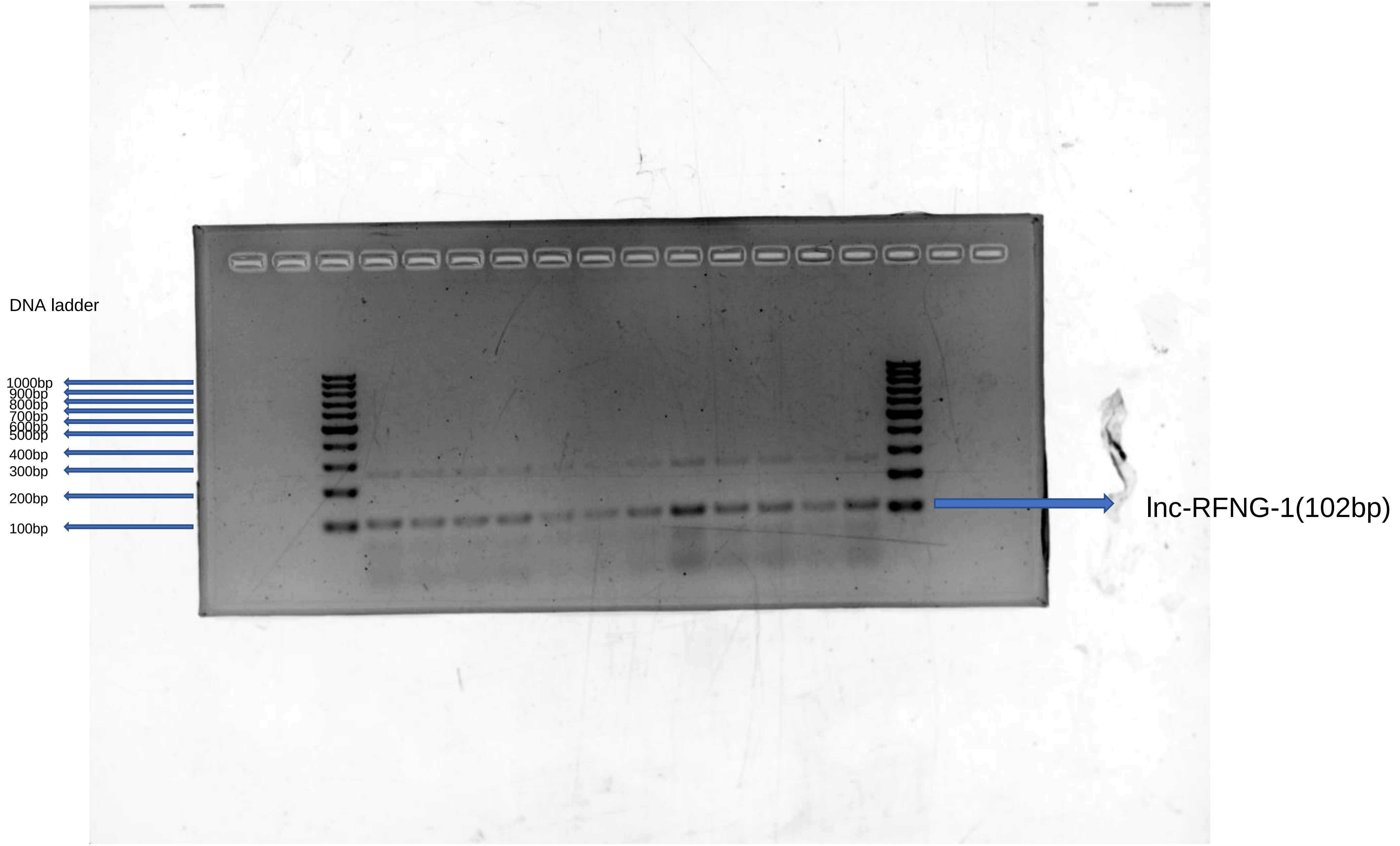
DNA ladder
1000bp
900bp
800bp
700bp
600bp
500bp
400bp
300bp
200bp
100bp
lnc-RFNG-1(102bp)

E

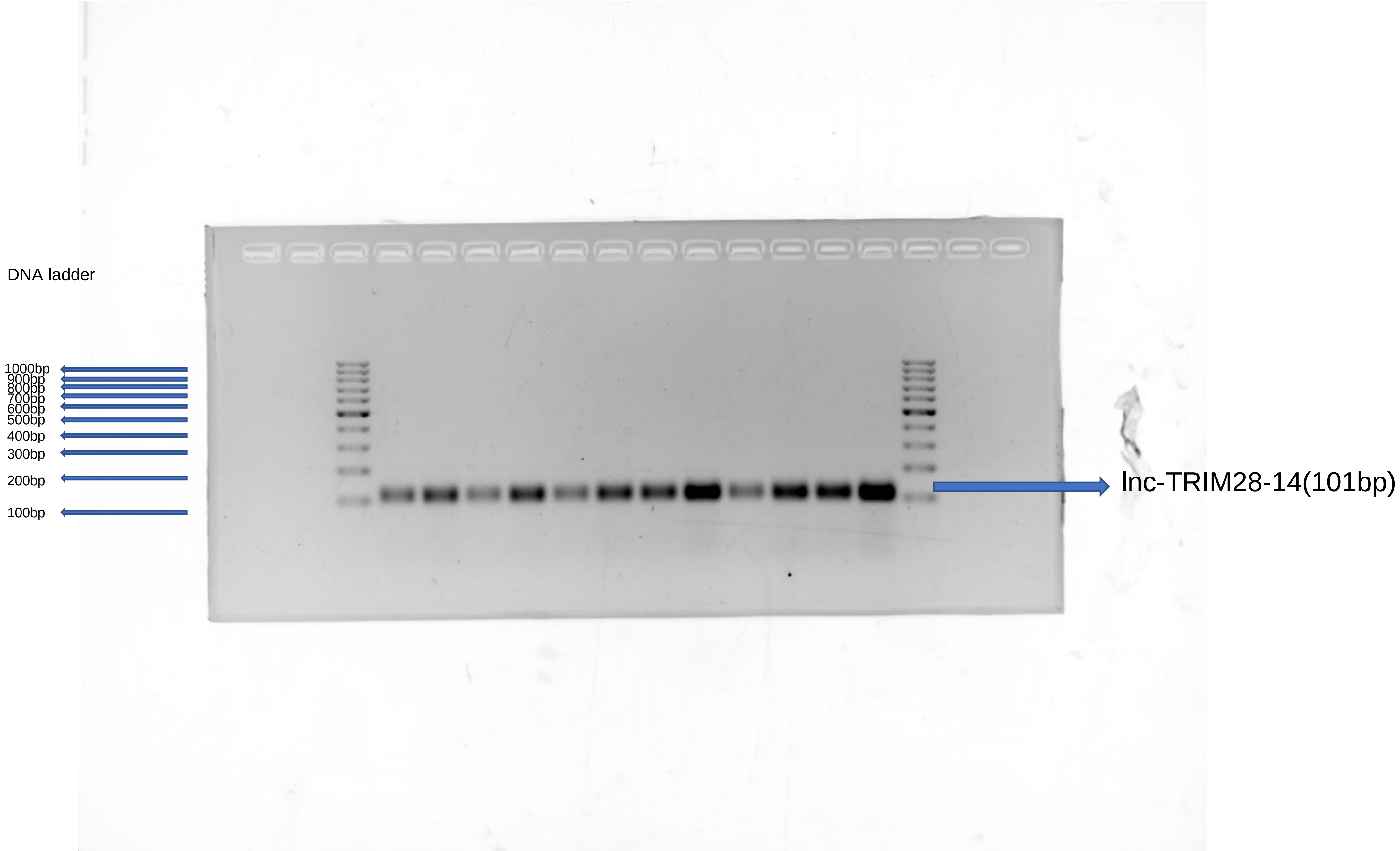
DNA ladder
1000bp
900bp
800bp
700bp
600bp
500bp
400bp
300bp
200bp
100bp
lnc-TRIM28-14(101bp)

F

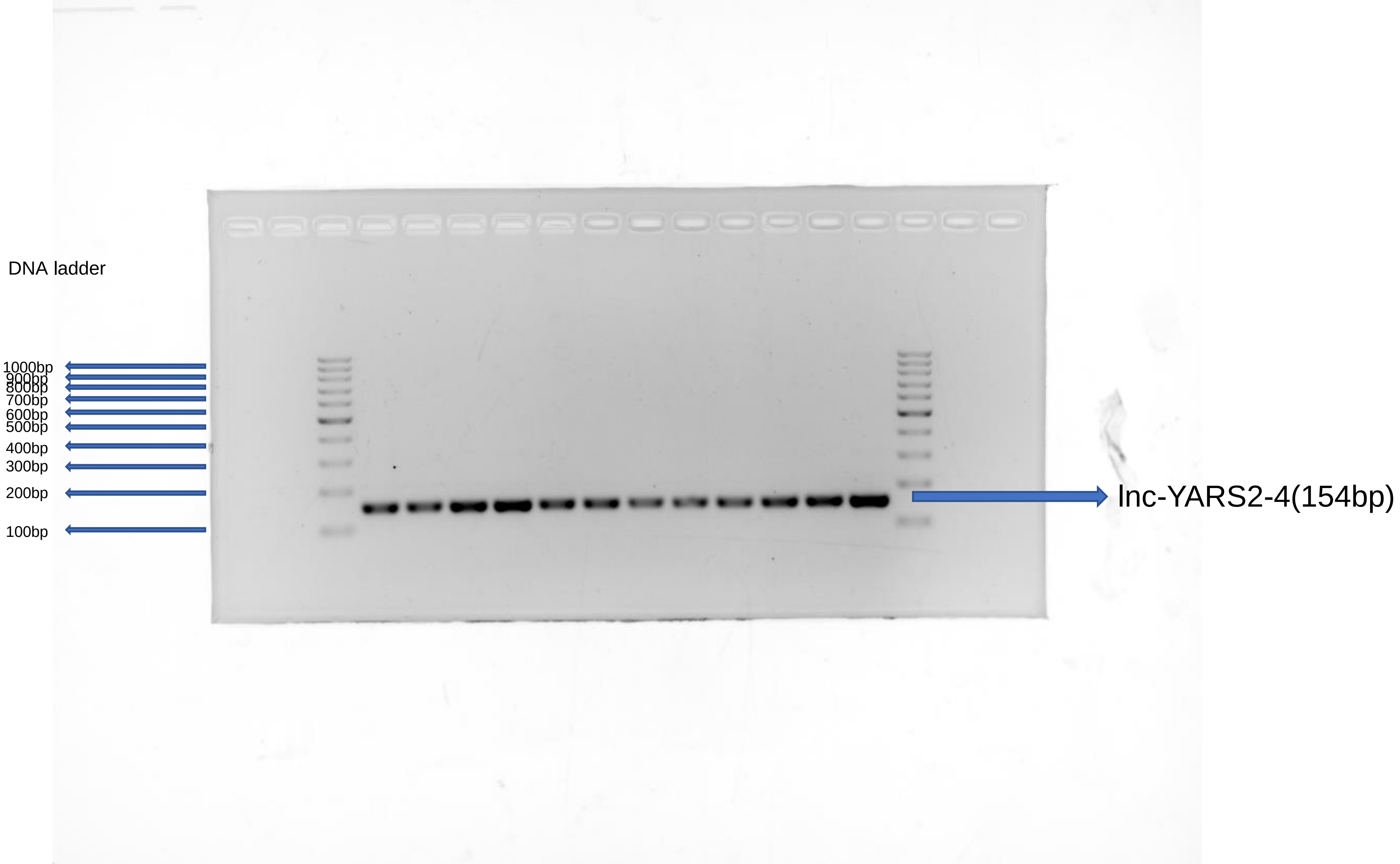

DNA ladder
1000bp
900bp
800bp
700bp
600bp
500bp
400bp
300bp
200bp
100bp
lnc-YARS2-4(154bp)

G

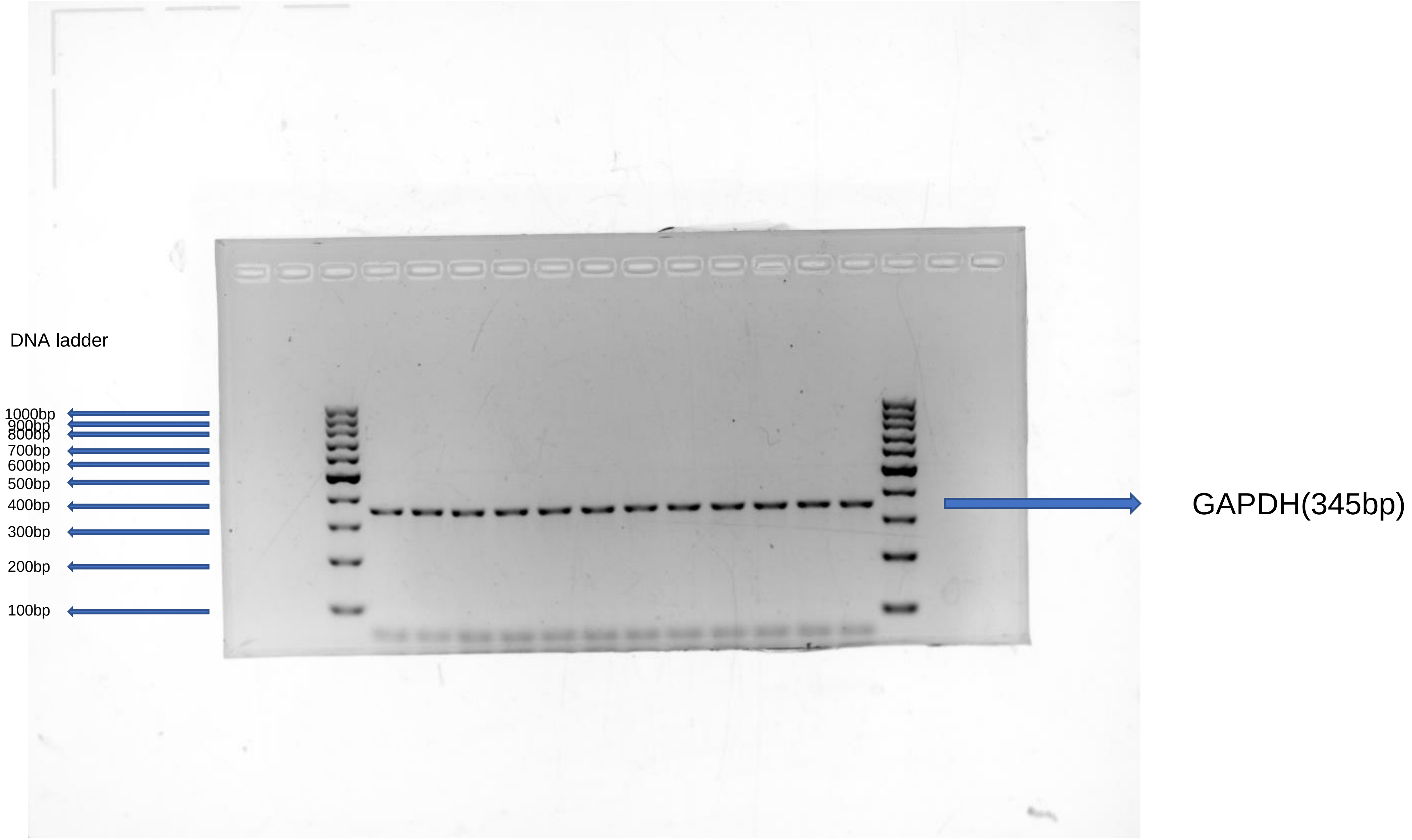
DNA ladder
1000bp
900bp
800bp
700bp
600bp
500bp
400bp
300bp
200bp
100bp
GAPDH(345bp)

Figure S4 Raw agarose gel electrophoresis images for hub lncRNAs analysis.

(A-G) The PCR products were analyzed with agarose gel electrophoresis on a 2% agarose gel, and expression of six hub lncRNAs and GAPDH was shown. The 12 lanes were N1, T1, N2, T2, N3, T3, N4, T4, N5, T5, N6, T6. The first 6 samples were derived from paracancerous (N) and cancerous (T) tissues from 3 gastric cancer patients without peritoneal metastasis, and the last 6 samples were derived from paracancerous (N) and cancerous (T) tissues from 3 gastric cancer patients with peritoneal metastases.
