# Supplementary material for: Identification and validation of crucial lnc-TRIM28-14 and hub genes promoting gastric cancer peritoneal metastasis
Source: BMC Cancer. 2023 Jan 23;23:76. doi: 10.1186/s12885-023-10544-8 (PMC9872371; doi:10.1186/s12885-023-10544-8)
Supplement: Supplementary file 13 — Additional file 13: Figure S5. Further verification of GCPM-related genes. (A-C) CD93, COL3A1 and COL4A1 expression in different gastric cancer tissues determined by qRT-PCR. (PT, primary tumors without GCPM; MT, primary tumors with GCPM; PM, GC peritoneal metastases)Student's t test, *, p < 0.05, **, p < 0.01, ***, p < 0.001, ****, p < 0.0001, ns, not significant. [file 12885_2023_10544_MOESM13_ESM.pdf]

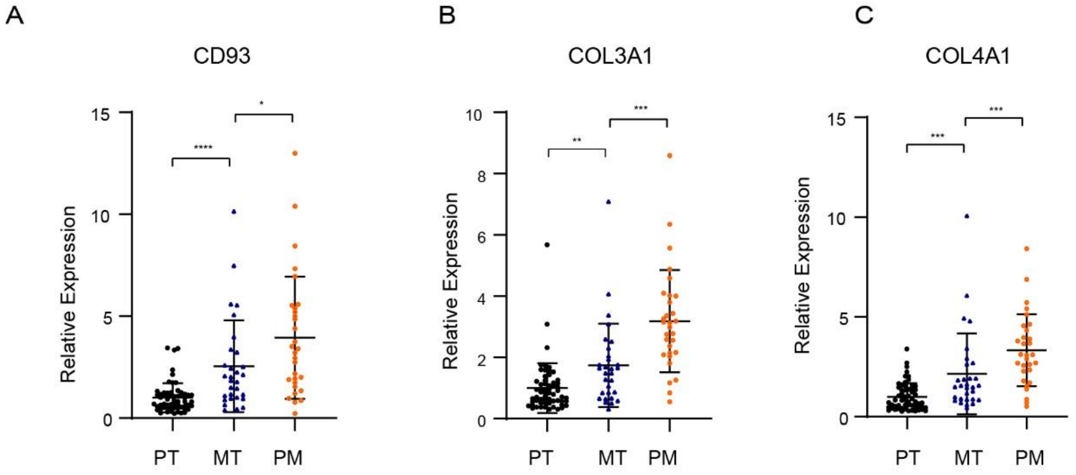

Figure S5. Further verification of GCPM-related genes. (A-C) CD93, COL3A1 and COL4A1 expression in different gastric cancer tissues determined by qRT-PCR. (PT, primary tumors without GCPM; MT, primary tumors with GCPM; PM, GC peritoneal metastases) Student's t test, *, $p < 0.05$, **, $p < 0.01$, ***, $p < 0.001$, ****, $p < 0.0001$, ns, not significant.
