# Supplementary material for: Identification and validation of crucial lnc-TRIM28-14 and hub genes promoting gastric cancer peritoneal metastasis
Source: BMC Cancer. 2023 Jan 23;23:76. doi: 10.1186/s12885-023-10544-8 (PMC9872371; doi:10.1186/s12885-023-10544-8)
Supplement: Supplementary file 10 — Additional file 10: Figure S2. Correlation analysis of hub lncRNAs and genes. (A) The lncRNA-mRNA correlation network. Based on the correlation coefficient > 0.6, p < 0.05 as the cut-off values, the closer the distance is, the higher the correlation coefficient. (B) Venn analysis for co-expressed genes of lnc TRIM28-14 and genes in magenta module. (C) Venn analysis showing that 6 candidate genes exactly included in the intersection in B. (D) PCA plot showing the correlation between GCPM related biomarkers. [file 12885_2023_10544_MOESM10_ESM.pdf]

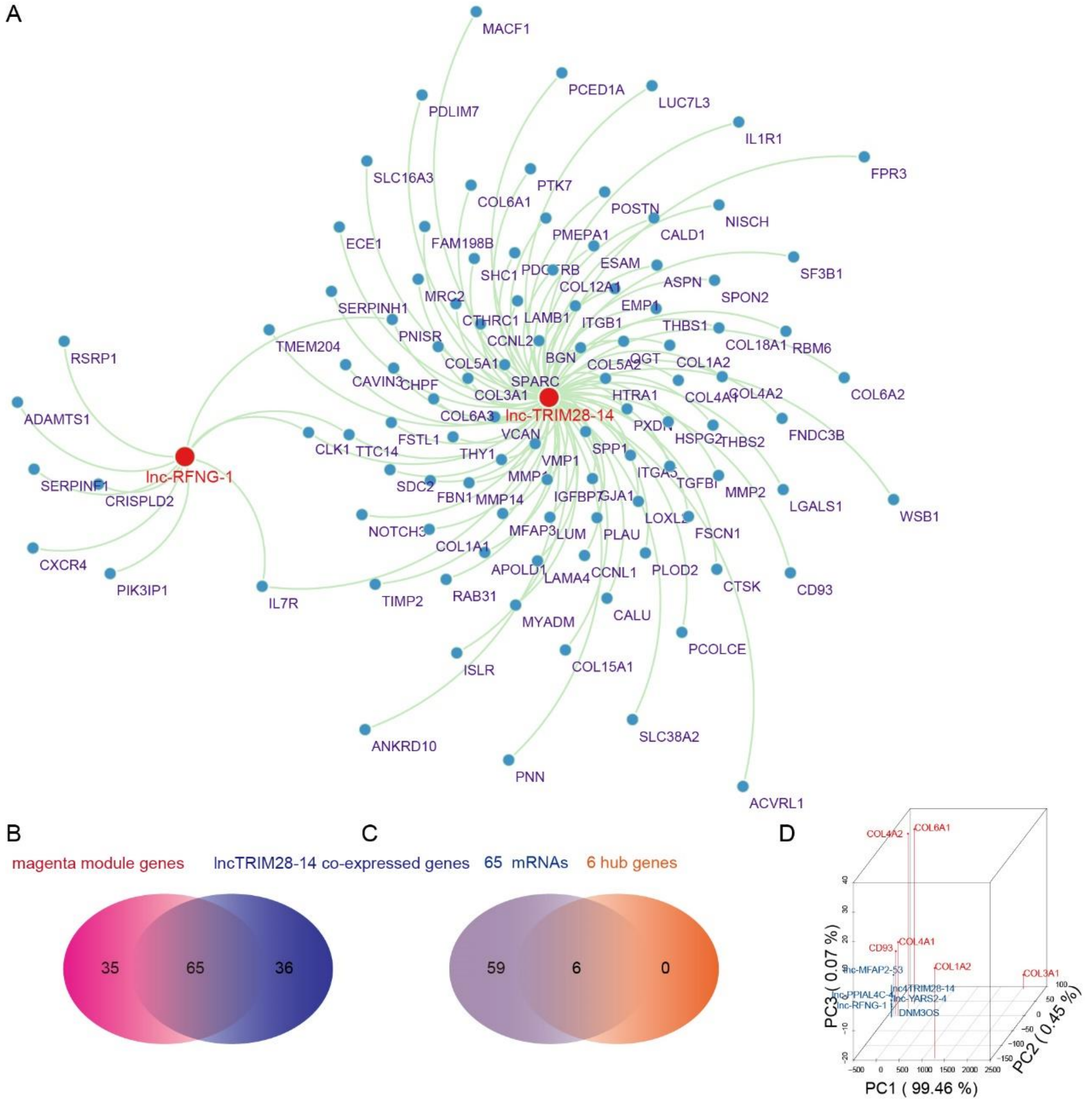

Figure S2 Correlation analysis of hub lncRNAs and genes. (A) The lncRNA-mRNA correlation network. Based on the correlation coefficient > 0.6, $p < 0.05$ as the cut-off values, the closer the distance is, the higher the correlation coefficient. **(B)** Venn analysis for co-expressed genes of lnc-TRIM28-14 and genes in magenta module. **(C)** Venn analysis showing that 6 candidate genes exactly included in the intersection in **B**. **(D)** PCA plot showing the correlation between GCPM-related biomarkers.
